# Unexpected hosts: imaging parasitic diseases

**DOI:** 10.1007/s13244-016-0525-2

**Published:** 2016-11-23

**Authors:** Pablo Rodríguez Carnero, Paula Hernández Mateo, Susana Martín-Garre, Ángela García Pérez, Lourdes del Campo

**Affiliations:** 1 Radiology Department, La Princesa University Hospital, Diego de León 62, 28006 Madrid, Spain; 2 Radiology Department, Clínico San Carlos University Hospital, Prof. Martín Lagos s/n, 28040 Madrid, Spain; 3 Radiology Department, Gregorio Marañón University Hospital, Dr. Esquerdo 46, 28007 Madrid, Spain

**Keywords:** Parasitic diseases, Radiology, Ultrasound, Multidetector computed tomography, Magnetic resonance imaging

## Abstract

Radiologists seldom encounter parasitic diseases in their daily practice in most of Europe, although the incidence of these diseases is increasing due to migration and tourism from/to endemic areas. Moreover, some parasitic diseases are still endemic in certain European regions, and immunocompromised individuals also pose a higher risk of developing these conditions. This article reviews and summarises the imaging findings of some of the most important and frequent human parasitic diseases, including information about the parasite’s life cycle, pathophysiology, clinical findings, diagnosis, and treatment. We include malaria, amoebiasis, toxoplasmosis, trypanosomiasis, leishmaniasis, echinococcosis, cysticercosis, clonorchiasis, schistosomiasis, fascioliasis, ascariasis, anisakiasis, dracunculiasis, and strongyloidiasis. The aim of this review is to help radiologists when dealing with these diseases or in cases where they are suspected.

Teaching Points

• *Incidence of parasitic diseases is increasing due to migratory movements and travelling.*

• *Some parasitic diseases are still endemic in certain regions in Europe.*

• *Parasitic diseases can have complex life cycles often involving different hosts.*

• *Prompt diagnosis and treatment is essential for patient management in parasitic diseases.*

• *Radiologists should be able to recognise and suspect the most relevant parasitic diseases.*

## Introduction

Parasites are organisms that live in another organism at the host’s expense, sometimes causing harm and disease in the host itself. Many parasites have complex life cycles with different stages, some involving intermediary hosts besides the final host where the mature or adult form of the parasite lives.

The human body can be the definitive, intermediate, or accidental host of several different parasites including protozoa, helminths, arthropods, insects, amongst others. Several human parasites pose a considerable health problem in endemic areas, usually affecting the less developed regions of the world [1].

Although relatively uncommon in our daily practice in Europe, some of these parasitic diseases have been recently increasing in incidence due to immigration from endemic regions and burgeoning tourist travelling to these areas. Moreover, some parasitic diseases are still endemic in some European regions. Immunocompromised patients also are at a higher risk of becoming affected by parasitic diseases and can develop more virulent forms of these conditions.

Parasitic diseases can pose a diagnostic challenge and sometimes may not be included in the initial differential diagnosis. They can be mistaken and simulate other conditions as infections by other agents, non-infectious inflammatory disorders, or neoplastic processes. A good clinical setting is essential to suspect parasitic diseases. Specific and advanced imaging techniques for instance those of magnetic resonance (e.g. perfusion and diffusion-weighted images, spectroscopy, cholangiography sequences, hepatobiliary contrast agents), or combined radiology-nuclear medicine tests, such as positron emission tomography-computed tomography (PET-CT), can be helpful in solving the differential diagnosis, especially when parasitic disease mimics malignant neoplasms [2–8].

Radiologists may unexpectedly face some of these parasitic diseases in their practice, hence it is important to be familiar with some of its typical imaging findings. It is also helpful to understand the basic physiopathology of the main human parasitic diseases and remember their main clinical findings in order to achieve a correct diagnosis through a good clinical-radiological correlation, which leads to a prompt and appropriate treatment of these patients.

## Material and methods

We elaborated a list of some of the most prevalent human parasitic diseases that, although quite infrequent in Europe, most probably could be encountered in our daily practice as radiologists (Table 1). We searched and reviewed all the patients admitted to our institutions with any of these parasitic diseases from January 2002 to January 2016, when our radiology departments had a PACS (Picture Archiving and Communication System). In those cases in which we did not find representative images, we had to expand our search before 2002. We retrospectively examined all imaging tests performed in these patients in the PACS (Picture Archiving and Communication System) of our institutions (including radiographs, fluoroscopy examinations, ultrasound, computed tomography, and magnetic resonance). We selected representative images of each parasitic disease. We reviewed the main imaging findings of these diseases, life cycle of the parasites, signs and symptoms, diagnosis, and treatment, as described in the scientific literature.

**Table:** Table 1

Parasite	Disease	Transmission & host	Most affected organs/systems	Characteristic imaging findings
Protozoa				
Plasmodium spp (P.falciparum, P. vivax, P. malariae, P. ovale)	Malaria	Vector (Anopheles mosquito) *Human final host*	Systemic disease	Hepatosplenomegaly Diffuse cerebral oedema Cerebral infarcts T2 hyperintensity in cortex, basal ganglia, and cerebellum ARDS
Entamoeba histolytica	Amoebiasis	Fecal-oral (ingestion of cysts) *Human final host*	Gl tract Liver	Liver abscess
Toxoplasma gondii	Toxoplasmosis	Fecal-oral (ingestion of cysts from cat faeces) Foodborne (ingestion of cyst-containing meat) Vertical (mother-foetus) *Human final host*	Systemic disease CNS	Cerebral ring enhancing lesions-”asymmetric target sign”
Trypanosoma cruzi	American trypanosomiasis or Chagas disease	Vector (Triatomine bugs) *Human final host*	Gl tract Heart Nervous system	Megaoesophagus and megacolon Myocardiopathy
Leishmania spp	Leishmaniasis	Vector (Phlebotomus sandflies) *Human final host*	Liver-spleen (kala-azar) Skin. Mucocutaneous	Hepatosplenomegaly Lymphadenopathy
Cestodes (tapeworms)				
Echinococcus spp (E. granulosus, E. multilocularis, E. vogeli, E. oligarthrus)	Echinococcosis or hydatid disease	Fecal-oral (ingestion of eggs) *Human accidental intermediate host*	Liver Lungs	Hydatid cysts
Taenia solium	Cysticercosis and Taeniasis	Cysticercosis: Fecal-oral (ingestion of eggs) *Human accidental intermediate host*	Cysticercosis: CNS	CNS cysts-”cyst with dot sign” CNS nodular calcifications
Trematodes (flukes)				
Clonorchis sinensis (also Opisthorchis viverrini and O. felineus)	Clonorchiasis	Foodborne (ingestion of cyst-containing fish) *Human final host*	Biliary system	Dilated intrahepatic bile ducts
Schistosoma spp	Schistosomiasis or bilharzia or snail fever	Direct contact (through skin) *Human final host*	Gl tract and liver (portal venous system) GU system (paravesical venous plexus)	Gl: Chronic liver disease signs Portal hypertension signs Periportal cuffing *S. japonicum*: liver capsule “turtle back” GU: Linear calcifications of urinary bladder and distal ureter walls
Fasciola spp (F. hepatica, F. gigantica)	Fascioliasis or liver rot	Foodborne (ingestion of parasite-contaminated vegetables or water) *Human final host*	Liver Biliary system	Subcapsular liver lesions (linear, nodular, clustered) Bile duct dilatation Hepatic subcapsular haematoma
Nematodes (roundworms)				
Ascaris lumbricoides	Ascariasis	Fecal-oral (ingestion of eggs) *Human final host*	Gl tract Biliary system Lungs	Adult worms inside bowel lumen or biliary tree Lungs: Patchy ground-glass infiltrates
Strongyloides stercolaris	Strongyloidiasis	Direct contact (through skin) Autoinfection *Human final host*	Lungs and bronchi Gl tract	Lungs: Miliary nodules, interstitial infiltrates, alveolar infiltrates Bowel wall oedema
Dracunculus medinensis	Dracunculiasis or Guinea worm disease	Foodborne (ingestion of water contaminated with parasite-infected water fleas) *Human final host*	Subcutaneous tissues	“Worm-like" calcifications in soft tissues
Anisakis spp	Anisakiasis	Foodborne (ingestion of worm-containing fish) *Human accidental intermediate host*	Gl tract	Bowel wall submucosal oedema Oedema of Kerckring’s folds Ascites

## Malaria

### The parasite, its cycle, and human infection

Malaria is probably the most devastating parasitic disease in the world. The 2014 report from the World Health Organization (WHO) estimated that about 200 million people had been infected in the previous year causing near one million deaths. This disease is distributed in endemic tropical and subtropical areas in Africa, South America, and South Asia. It is secondary to infection by plasmodium protozoa, mainly P. falciparum or P. vivax, and less likely P. malariae or P. ovale. Transmission occurs from person to person by the bite of the female Anopheles mosquito. After reaching the bloodstream these parasites grow within the erythrocytes and are released by cyclic haemolysis.

The main symptom of malaria is episodic fever [9–11]. Abdominal signs and symptoms of malaria are usually mild and nonspecific and include abdominal pain or hepatosplenomegaly [9, 10]. The neurological manifestations are nonspecific as well, but cerebral malaria can progress rapidly having a high mortality rate (15–40 %) [9, 11, 12]. The primary thoracic manifestation of malaria is adult respiratory distress syndrome (ARDS) [9, 13].

### Imaging findings

**Fig. 1:**
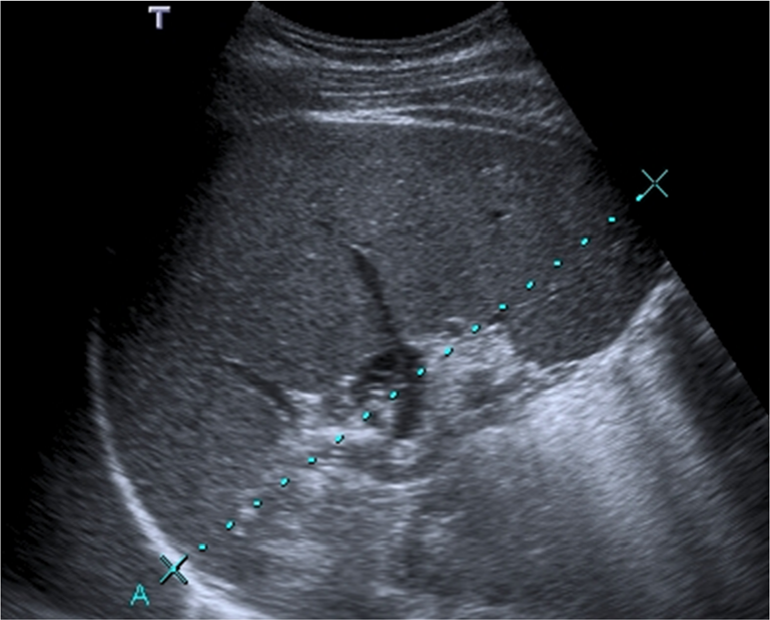
32-year-old patient, recently travelling from Cameroon and with previous history of malaria, presenting with fever, chills, generalised abdominal pain, and myalgia. The image of an abdominal US scan depicts splenomegaly of 17 cm. Plasmodium falciparum was detected in blood exams (the parasite was seen in thick blood smears, and antigens were also positive)

Imaging findings of malaria are often nonspecific and usually need a high clinical suspicion to relate them to the plasmodium infection. Abdominal findings consist of hepatoesplenomegaly with periportal oedema, gallbladder wall thickening, and ascites (Fig. 1). Splenic infarction and rupture have also been reported [9, 10]. Central nervous system (CNS) infection is usually caused by P. falciparum, a more aggressive form of malaria. It can manifest with different degrees of affection, ranging from a normal brain (30-50 % cases) to diffuse cerebral oedema with or without focal infarcts [14]. Petechial haemorrhages can be seen on magnetic resonance (MR) as high signal foci on T1-weighted images (T1WI) and small foci of low signal on T2*-weighted images. T2-weighted images (T2WI) demonstrates hyperintensity in the cortex, basal ganglia, and cerebellum [9, 11–13, 15]. The primary thoracic manifestation is ARDS consisting of diffuse interstitial oedema. Pleural effusions and lobar consolidations may also be seen, usually secondary to P. falciparum and associated with mortality up to 80 % [9, 16].

### Diagnosis and treatment

Diagnosis is made by the identification of trophozoites in thick or thin blood smears. Antigens can also be detected in blood. As this disease has no specific radiological findings, it can only be suggested by radiologists under a very high clinical suspicion, as in febrile patients returning from endemic areas.

Malaria is treated with specific antimalarial antibiotics (e.g. quinine, chloroquine), together with supportive therapy and anticonvulsant drugs in case of seizures [9, 17].

## Amoebiasis

### The parasite, its cycle, and human infection

Amoebiasis is widely spread all over the world, but is more prevalent in India, the Far East, Africa, and Central and South America. It is mainly caused by the Entamoeba histolytica protozoa. Other amoeboids such as Naegleria fowleri, Acanthamoeba astronyxis, and Balamuthia mandrilaris can also cause human infections [9]. Humans become infected by ingestion of contaminated food or water containing amoebic cysts [4, 9, 18, 19]. These cysts become trophozoites that invade the bowel wall mucosa entering the portal circulation [9, 18].

 E. histolytica infects the gastrointestinal tract sometimes remaining in a latent stage for many years, but other times it can cause more aggressive forms of the disease ranging from colitis to dysentery, or liver, thoracic, and more rarely brain abscesses and meningoencephalitis [4, 9, 18–20].

### Imaging findings

**Fig. 2:**
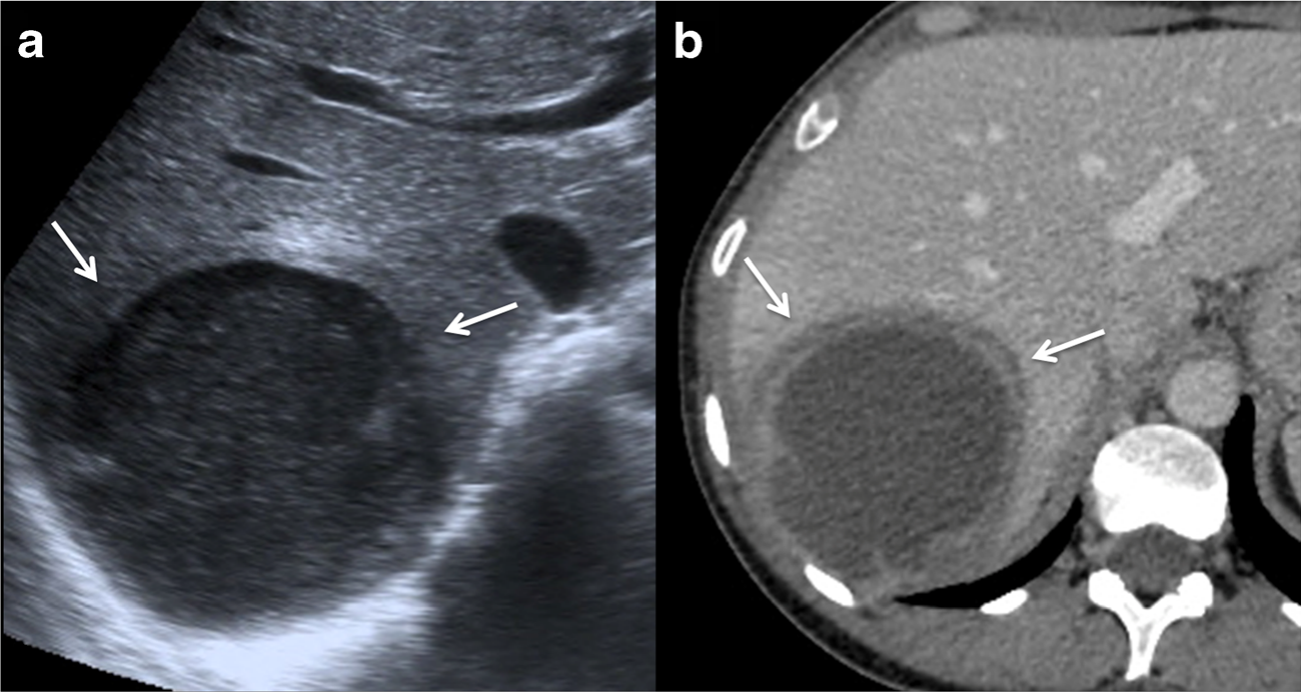
Amoebic liver abscess. **a** Axial US image shows a rounded, well-defined hypoechoic lesion with thick walls, located in the right hepatic lobe (*arrows*). Note the posterior acoustic enhancement that prompts a partially cystic nature. **b** Axial image of a contrast-enhanced CT from the same patient as in figure a, showing a large cystic mass with solid walls in the right hepatic lobe. Note the enhanced thickened wall of the lesion and perilesional oedema (*arrows*). The patient had positive serology for E. histolytica (IgG antibodies), and amoebic cysts were found in the faeces. The patient was treated successfully with amoebicidal drugs with complete resolution of the liver abscess

Amoebic liver abscess is the most frequent extraintestinal complication of amoebiasis, and its imaging features are sometimes indistinguishable from a pyogenic abscess; however, epidemiologic and clinical features, as well as positive amoebic antibodies, help to make the diagnosis. Ultrasound scans (US), computed tomography (CT), and MR are useful to detect these liver lesions. These abscesses tend to be located near the capsule and usually have an enhancing thick wall with perilesional oedema [4, 9, 18, 21] Fig. 2.

Thoracic infection is the second most common manifestation of extraintestinal amoebiasis and may result mainly from direct extension from an hepatic abscess to the thorax or by haematogenous spread, with pleural effusion the most common manifestation, followed by lung consolidations. There can be a fistula from the liver infection to the airway through the diaphragm, leading to an hepatobronchial or bronchobiliary fistula [9, 18].

Imaging of amoebic meningoencephalitis is nonspecific and has rarely been described. In healthy patients it can cause a primary form of meningoencephalitis (PAM) with a fulminant course in most cases and a mild long-term form in immunocompromised patients called granulomatous meningoencephalitis (GAE) [9, 20, 22]. In CT, GAE may show multifocal enhancing lesions in the cortex and brainstem. In MR small hyperintense lesions on T2WI can be seen with heterogeneous or ring-like pattern of enhancement. PAM may show a brain pattern of oedema and hydrocephalus, with rapid progression of the disease [20].

### Diagnosis and treatment

Since imaging features of amoebic disease are quite unspecific, diagnosis is made on culture, serology, or immunofluorescence on biopsy specimens. Amoebic cysts can be found in the faeces. The classic anchovy paste-like material can be obtained from an amoebic abscess. After the diagnosis is confirmed amoebicidal therapy such as metronidazole or ketoconazole should be started, as it is highly effective. Catheter drainage of abscesses performed with imaging guidance is sometimes performed.

## Toxoplasmosis

### The parasite, its cycle, and human infection

Toxoplasmosis is the most prevalent parasitic disease worldwide, and it is caused by Toxoplasma gondii, which is a protozoan affecting one third of the total global population. It is more frequent in the tropics and in warm areas where cats are numerous. The T. gondii life cycle has two differentiated phases. The so-called sexual phase takes places in felines, which are the parasite’s primary host. The following asexual phase can occur in other warm-blooded animals, including humans [8, 9, 23].

Infection in humans can happen by three means: fecal-oral transmission through the ingestion of an infected cat’s faeces (usually through contaminated fruits and vegetables), foodborne through the ingestion of toxoplasma cysts in poorly cooked meat (mainly pork, lamb, or venison), or vertical transmission from the mother to the foetus. Toxoplasma cysts are mostly found in skeletal muscle, heart, and brain, and they can remain latent or course subclinically in healthy adults [8, 23]. Foetal infection can cause congenital toxoplasmosis coursing with a wide spectrum of manifestations, from mild symptoms that can remain unapparent until late in infancy, to more fatal and severe forms in newborns that are now rare thanks to preventive and protective measures during pregnancy. The most affected group of population by toxoplasmosis are immunocompromised individuals such as AIDS and bone marrow transplanted patients often developing more aggressive forms of the disease, especially in the CNS [8, 9, 24].

### Imaging findings

This parasite infects the CNS of approximately 10 % of AIDS or immunocompromised patients. Most lesions are located in the basal ganglia, corticomedullary junction, white matter, and periventricular region due to its haematogenous spread [8, 9, 23, 24]. MR has proved to be the most sensitive imaging modality for the detection of cerebral toxoplasmosis, and it is able to delineate the true extent of the disease [8].

**Fig. 3:**
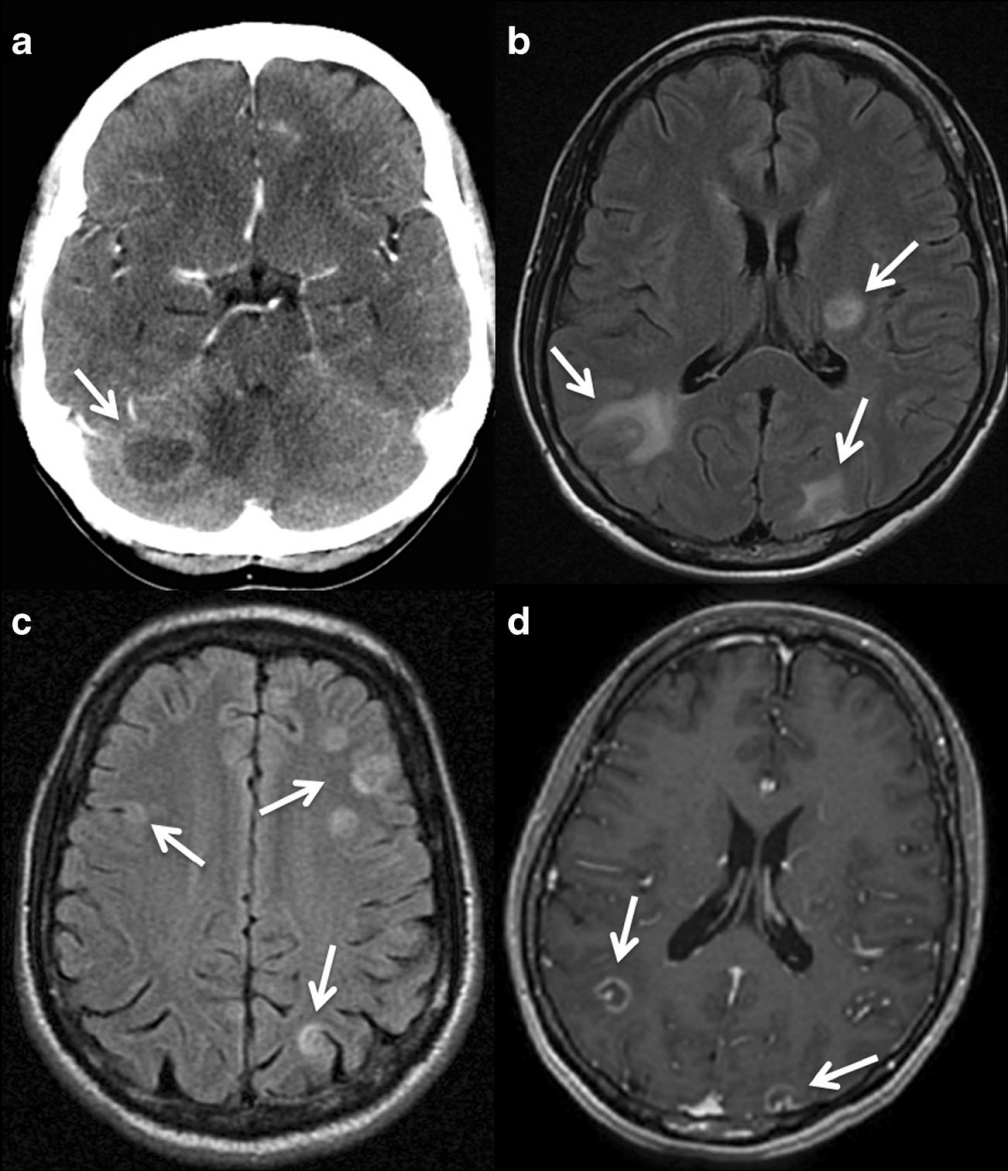
Intracranial toxoplasmosis in a 45-year-old patient with AIDS. **a.** Contrast-enhanced CT displays a hypoattenuating lesion with ring-enhancement in the right hemisphere of the cerebellum (*arrow*). **b** and **c** Axial fluid-attenuated inversion recovery MR images showing multiple brain lesions predominantly located in the grey-white matter junction, with mild mass-effect and some of them with surrounding oedema (*arrows*). **d** Gadolinium-enhanced axial T1-weighted MR image, the lesions (*arrows*) show ring-enhancement and a small eccentric nodule inside, the “asymmetric target sign,” which is suggestive of toxoplasmosis

Brain infection produces scattered lesions showing low attenuation on CT, low or high signal on T1WI (suggesting haemorrhagic components), and high signal on T2WI with surrounding oedema. Toxoplasmosis lesions show homogeneous nodular or ring enhancement (Fig. 3) [8, 9, 23–26]. In immunocompromised patients, enhancement may vary according to their cellular immunological response; if there is poor enhancement it may be minimal or absent. An imaging finding highly suggestive for toxoplasmosis is the “asymmetric target sign,” which is a small eccentric nodule along the wall of the enhancing ring, likely representing a thrombosed vein (Fig. 3d) [23, 25, 27]. Diffusion- and perfusion-weighted images (DWI and PWI) and spectroscopy can be valuable tools to differentiate toxoplasmosis from other mimicking conditions, such as brain lymphoma and other infections, particularly in immunocompromised patients. Toxoplasmosis can show peripheral restricted diffusion, while pyogenic abscesses typically show central restricted diffusion, PWI is low in toxoplasmosis and high in lymphoma and other neoplasms. Increased lipid-lactate peak in spectroscopy is characteristic of both toxoplasma and pyogenic abscesses [8, 28–30]. Thalium single-photon emission computed tomography and PET-CT usually show lower uptake in toxoplasmosis and higher in lymphoma [31–34].

The lungs and heart are the most frequently affected organs after the CNS. Radiologic manifestations are nonspecific and may be similar to other opportunistic diseases in immunocompromised patients. Diffuse bilateral reticulonodular opacities in the lungs are the most frequent thoracic finding [9].

In congenital infection, calcifications, hydrocephalus, and microcephaly are common [25, 26]. Other congenital findings are ascites and hepatosplenomegaly [26].

### Diagnosis and treatment

A positive antibody titre for T. gondii is not diagnostic of active toxoplasmosis, it only indicates that there has been contact with the parasite. Prevalence of toxoplasma seropositivity is very variable in Europe, ranging from 10 % up to 90 % in some regions [35]. Up to 20 % of patients with AIDS may not have detectable antitoxoplasma antibodies. Polymerase chain reaction testing (PCR) of peripheral blood samples has a high sensitivity and specificity for the diagnosis [24].

Concerning treatment, various antitoxoplasma agents are available such as pyrimethamine alone or combined with sulfadiazine [8, 9, 24]. Once toxoplasmosis is suspected by imaging criteria and serologic tests are positive, medication is begun and the response of treatment can be monitored with clinical examination and CT or MR.

## American trypanosomiasis or Chagas disease

### The parasite, its cycle, and human infection

 Trypanosoma cruzi is the aetiologic agent for the American trypanosomiasis or Chagas disease, and it is widely spread in Central and South America.

This parasite is acquired by humans when the vector of the parasite, an insect from the Triatominae family, bites the individual and defecates infected faeces on the skin [9, 18]. If the bite occurs near the eyes, periorbital oedema appears and is called Romaña’s sign. The indurated skin lesion is called “chagoma” in other regions of the body [36]. Scratching of the bite favours the inoculation of the parasites from the infected faeces. The parasites multiply within the macrophages, which eventually rupture and release amastigotes that enter the bloodstream invading diverse organs such as the heart and the gastrointestinal tract [18].

### Imaging findings

This disease has different stages. Acute manifestations are uncommon and may include fever, myocarditis, and rarely meningoencephalitis in immunocompromised individuals [9, 18, 37]. Later, in the subacute phase, hepatosplenomegaly and lymphadenopathy are the most common findings. Around 15-30 % of the infected individuals develop chronic forms of the disease, mainly affecting the heart, digestive tract, and nervous system.

**Fig. 4:**
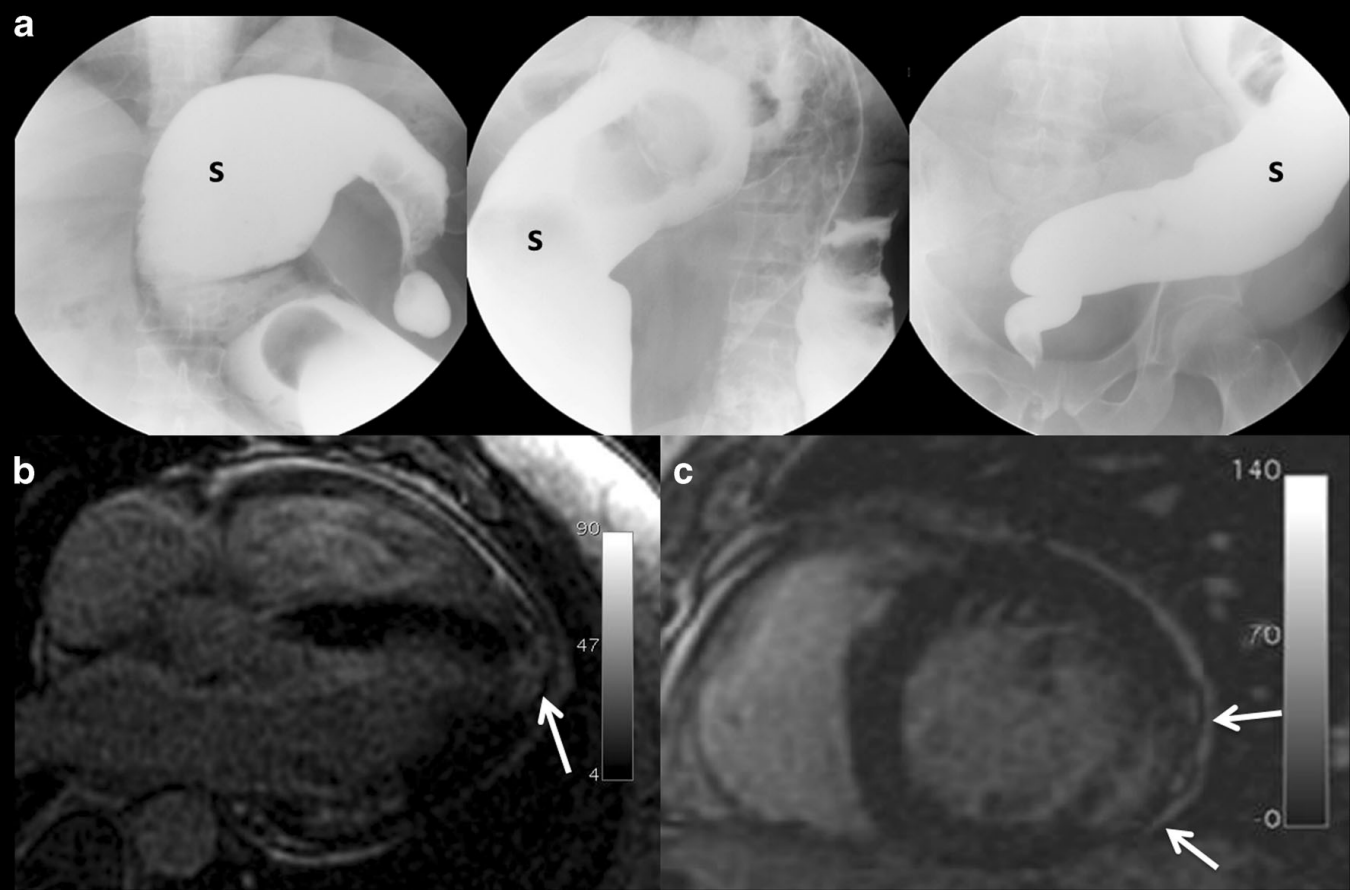
**a** Barium enema images of a 42-year-old patient native to Bolivia, it shows megacolon fundamentally at the expense of sigmoid colon (s). Chagas disease was confirmed by serologic testing. **b** and **c** Cardiac chronic Chagas disease. 50-year-old male from Bolivia. Cardiac MR delayed contrast-enhanced sequence with four-chamber view (**b**) and two-chamber short axis view (**c**); some areas of delayed transmural enhancement can be appreciated in the left ventricle (*arrows*) with a non-coronary territory distribution, indicating myocardial fibrosis or necrosis. Note the basal inferolateral and apical locations, the most frequently affected areas in cardiac chronic Chagas affection

Gastrointestinal compromise in the chronic form of the disease is related to damage to neurons of the myenteric plexuses, with development of megaoesophagus (chagasic achalasia) and megacolon [18]. Fluoroscopic studies (single- or double-contrast oesophagography and barium enema) usually confirm this condition showing massive dilatation of the oesophagus and colon (Fig. 4a) [9, 38].

Cardiac manifestations of chronic Chagas include chronic myocarditis with focal and diffuse affection and leading to fibrosis, myocardial necrosis, and atrophy. Myocardial fibrosis or necrosis is seen in MR as a characteristic delayed contrast enhancement after intravenous (iv) injection of gadolinium contrast with a transmural, subepicardial, or midwall distribution. The left ventricle inferolateral basal segments and the apical region are most commonly affected (Fig. 4b-c). Another characteristic feature of chronic Chagas disease is an apical aneurysm of the left ventricle with transmural delayed enhancement [9, 18, 39].

### Diagnosis and treatment

The diagnosis is usually made by detecting in the patient’s blood either the parasite or antibodies against it, where PCR is the most useful technique, especially in infected patients with borderline serology not detected by other methods [9, 18].

During the acute phase of Chagas disease, anti-Chagas drugs such as benzinidazole and nifurtimox may heal the disease. Unfortunately, once the disease reaches its chronic phase, antiparasitic drugs will not be able to cure it definitely, and treatment will be based on trying to palliate specific chronic conditions such as the cardiac or gastrointestinal affections [40].

## Leishmaniasis

### The parasite, its cycle, and human infection

Leishmaniasis is a disease striking the less developed areas of Southeast Asia, East Africa, and Latin America, but it is also endemic in several Mediterranean countries. Caused by Leishmania spp, there are three main types of leishmaniasis: visceral or kala-azar, which is the most deadly parasitic disease after malaria, cutaneous, and mucocutaneous. Leishmaniasis is transmitted by the bite of certain species of sand flies from the genus Phlebotomus. The increase of this disease in developed countries is related to immunodepression and the rise of international movements of people due to migration or tourism [9, 41].

### Imaging findings

**Fig. 5:**
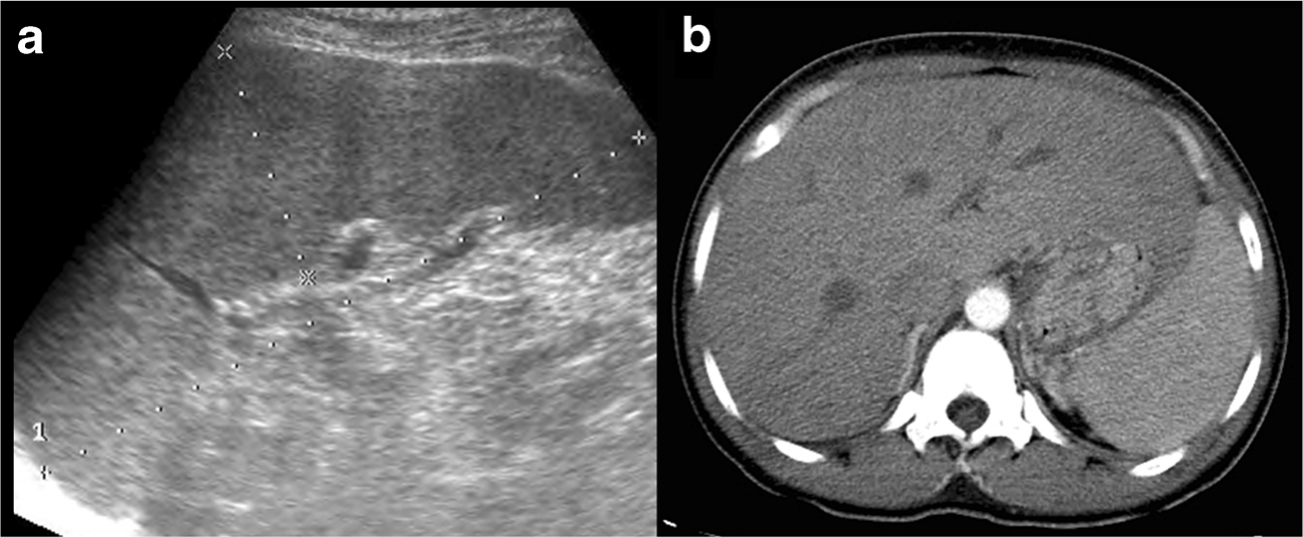
Visceral leishmaniasis. 52-year-old patient native to Peru, presenting with fever, weight loss, and pancytopenia. Serological exams confirmed leishmaniasis. **a** Sagittal US image shows splenomegaly. **b** Contrast-enhanced CT image shows hepatosplenomegaly

The imaging findings of leishmaniasis are nonspecific, usually consisting of hepatosplenomegaly and enlargement of lymph nodes (Fig. 5). Rarely, hepatic or splenic nodules are seen on visceral leishmaniasis, appearing in US as solid nodules in surrounded by a peripheral hypoechoic halo and hypovascular lesions on CT and MR [9].

### Diagnosis and treatment

The diagnosis can be established by serologic exam or skin testing of the leishmania antigen, and it can be confirmed by the detection of the intracellular parasite in liver, spleen, or bone marrow biopsy [9, 41].

After confirmation of the disease, appropriate treatment with liposomal amphotericin B is usually started. It is the most used drug in travellers returning from endemic areas [41].

## Echinococcosis or hydatid disease

### The parasite, its cycle, and human infection

Echinococcosis is caused by Echinococcus tapeworms, mainly E. granulosus (cystic echinococcosis) and less frequently E. multilocularis (alveolar echinococcosis). E. vogeli and E. oligarthrus are much less common. It is a worldwide infection endemic in herding and grazing regions of the Mediterranean, the Americas, Russia, Central Asia, and China [42].

Dogs and other canidae are the main definitive host, in whose small intestine lives the adult worm. Definitive hosts release the eggs of the parasite in their faeces and the intermediate hosts (usually sheep or goats) eat them becoming infected and developing hydatid cysts in different organs. The definitive host gets infected through the ingestion of intermediate hosts flesh infected with cysts.

Humans can accidentally become intermediate hosts after the ingestion of the worm’s eggs present in contaminated food, water, or soil, but usually the cycle of the Echinococcus ends in them. In the intestine the eggs release the embryos (onchospheres) which penetrate the bowel wall and enter portal or lymphatic circulation. The onchospheres usually settle in the liver, followed by the lung. Once in a solid organ the onchospheres turn into the larval stage forming the hydatid cyst, which can remain viable for years [9, 42].

### Imaging findings

There are two main classifications of echinococcal cysts based on US patterns of hepatic cysts that mostly apply to E. granulosus: the Gharby classification of 1981 and the WHO classification of 2003 [43, 44]. The WHO classification has also proved to be applicable in other locations and with other imaging techniques, for example in cerebral hydatid cysts evaluated with MR [45]. There is also a more simple radiological classification (from type I to type III cysts, where type IV are complicated cysts) [46, 47]. These classifications correlate with the progressive degeneration of the hydatid cysts Table 2.

**Table 2:**
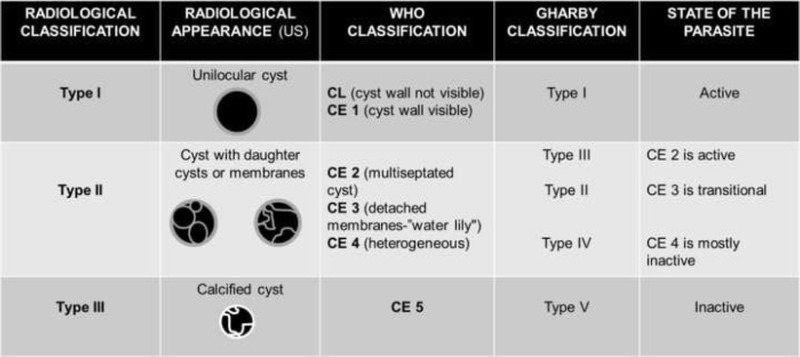
Classifications of the hepatic hydatid cysts and their imaging correlate

**Fig. 6:**
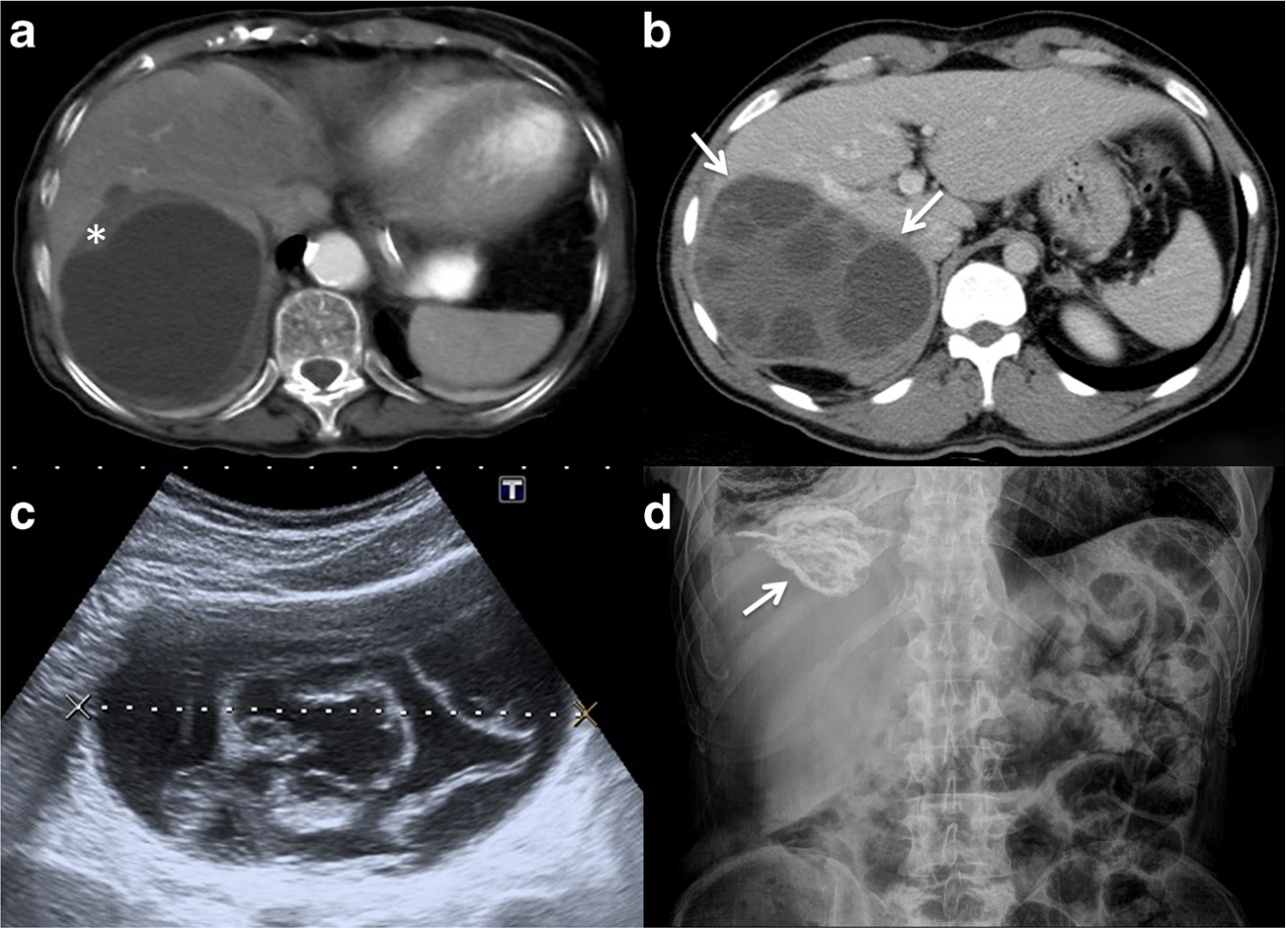
Hepatic echinococcal cysts. **a** Type I cyst (WHO CE 1). Contrast-enhanced CT image of a unilocular echinococcal cyst in the right hepatic lobe, note the thicker anterior wall (*asterisk*). **b** Type II cyst (WHO CE 2). Contrast-enhanced CT image of a multilocular echinococcal cyst in the right hepatic lobe containing multiple daughter cysts in the periphery and mimicking a “rosette” (*arrows*). **c.** Type II cyst with contained ruptured (WHO CE 3). Axial US image shows floating membranes (“water-lily sign”) inside a cyst, consistent with rupture of the endocyst or daughter cysts. **d** Type III cyst (WHO CE 5). Fragment of an abdominal radiograph that incidentally showed a calcified hepatic hydatid cyst (*arrow*)

Type I cyst of the radiological classification is the earliest stage Fig. 6a. They are unilocular cysts that can be confused with other simple cysts. Some features can help rising the suspicion, such as the presence of denser material in the cyst (“falling snowflakes” inside the cyst in the US, or higher attenuation contents in CT), thicker walls, or the presence of a low signal intensity rim in T2WI MR, likely representing that the pericyst is rich in fibrotic tissue [48, 49]. Type I cysts correspond to WHO classifications CL (when the cyst wall is not visible) and CE 1 (when the cyst wall is visible).

Type II cysts (WHO CE 2) contain daughter cysts in the periphery appearing as multiseptated cysts mimicking a “rosette”. Usually the mother cyst has a denser content than the daughter cysts that can be seen with a higher attenuation in CT and with different signal intensity in MR [47, 50] Fig. 6b. Contained ruptures of the cyst due to trauma, treatment or spontaneous degeneration appear as floating membranes (“water-lily sign”), and are classified as WHO CE 3 (Fig. 6c) [49, 51]. The presence of calcifications in the cysts correlates with its degeneration, but unless the cyst is completely calcified it does not imply that all larvae are necessarily dead. WHO stage CE 4 is between type II and type III cysts; they are degenerating cysts that are heterogeneous and can have a solid appearance.

Type III cyst (WHO CE 5) are chronic hydatid cysts almost completely calcified, usually indicating that there are no more living parasites. Calcifications are typically curvilinear. Some calcified cysts can be incidentally discovered in asymptomatic patients usually on CT or even on conventional radiographs (Fig. 6d) [48, 49, 52].

**Fig. 7:**
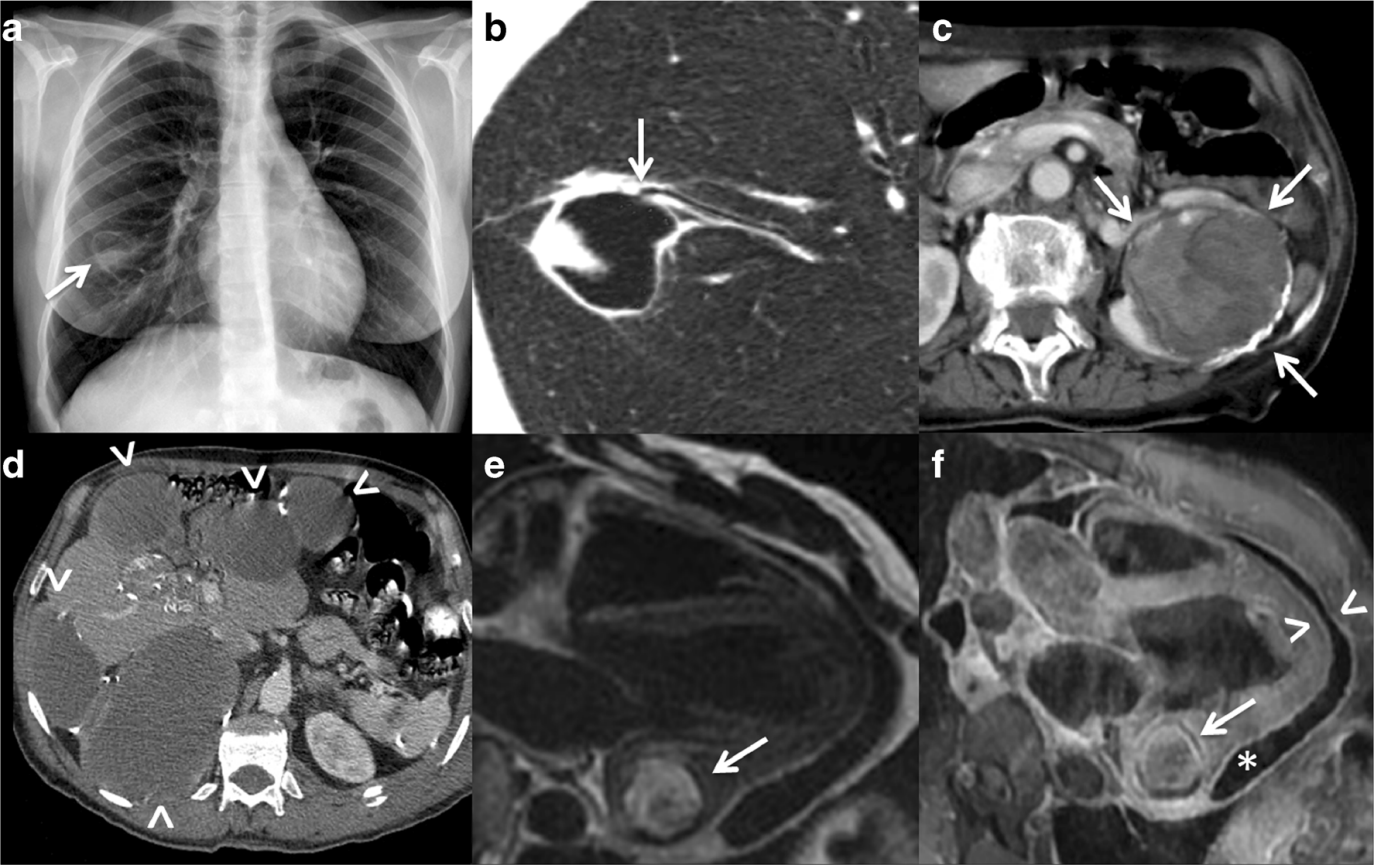
Echinococcosis. **a** Chest radiograph. There is a cystic lesion in the right lower lobe (*arrow*) with an air-fluid level corresponding to a pulmonary hydatid cyst communicated with the airway. The patient had expectoration of cyst components. **b** CT image of the same patient as in figure a, showing the hydatid cyst communicating with a subsegmental bronchus (*arrow*). **c** Contrast-enhanced CT image showing a complex lesion in the left kidney (*arrows*) containing dense material and membranes, and having a partially calcified wall, it proved to be a renal hydatid cyst surgically and histopathologically. **d** Multiple cystic lesions in the liver and peritoneum, some with partially calcified walls, which also proved to be hydatid disease surgically and histopathologically (*arrowheads*). **e** T2-weighted double inversion recovery MR image of the heart. It shows a hyperintense intramyocardial lesion in the posterobasal segment of the left ventricle that proved to be a hydatid cyst surgically and histopathologically (*white arrow*) **f** Gadolinium-enhanced fat-saturated T1-weighted double inversion recovery MR image of the heart, from the same patient as in **e**. It shows subtle peripheral enhancement of the lesion intramyocardial lesion (*arrow*), and pericardial effusion (*asterisk*) with pericardial enhancement (*arrowheads*) indicating pericarditis

Lungs are the second most common organ of hydatid affection in adults, and arguably the most frequent place of infection in children. Because the lungs have parenchymal architecture, the cysts can reach bigger dimensions there and can destroy surrounding bronchi establishing communication with the airway and leading to the expectoration of cyst components by the patient and the filling of the cyst with air (Fig. 7a–b). Transdiaphragmatic infective route from the liver can also occur [47, 49, 53].

Hydatid cysts in other locations are rare, being the kidneys and peritoneum one the most frequent sites. The imaging appearance of hydatid cysts in other solid organs is similar to the hepatic hydatid cysts. Peritoneal seeding is most of the times secondary to ruptured hepatic cysts either spontaneously or during an interventional or surgical procedure Fig. 7c–f.

E. multilocularis infection is much less common. The lesions tend to be more invasive mimicking neoplasms. Its cysts are typically multiloculated resembling alveoli, hence the name of alveolar echinococcosis [4, 47]. Accurately distinguishing the different types of Echinococci species by imaging can be difficult.

### Diagnosis and treatment

Radiologist can be the first ones to detect hydatid infection, sometimes incidentally in asymptomatic patients. Imaging can also help classifying the stage of the cysts and therefore hint its activity. Exact diagnosis of the infecting species is given by serology, histopathology, or immunohistochemistry.

Traditional standard treatment for hydatid cysts has been surgical removal combined with antiparasitic drugs (mainly albendazole or mebendazole). Percutaneous drainage of the cysts (puncture, aspiration, injection of scolicidal agent, re-aspiration) can also be performed by radiologists in selected patients, especially in non-complicated type I and type II cysts or in patients that are not suitable for surgical treatment [38–41].

## Cysticercosis

### The parasite, its cycle, and human infection

Cysticercosis is caused by Taenia solium, also known as pork tapeworm. It poses a health problem especially in Latin America, sub-Saharan Africa, South-East Asia, China, and the Indian subcontinent [54].

 T. solium lives in the small intestine of humans, their definitive host. Their eggs are released with the faeces and ingested by pigs, the intermediate host. When pigs are infected they develop cysts in their soft tissues (cysticerci). The cycle is closed when the humans ingest raw or poorly cooked pork containing cysticerci and develop the adult tapeworm in their intestine (taeniasis) [55].

Cysticercosis happens when humans accidentally become the intermediate host by ingesting water, food, or soil contaminated with eggs of the parasite. The eggs release the embryos (onchospheres) in the bowel lumen, which penetrate the intestine wall entering portal circulation. The onchospheres will end up lodging in the capillaries of richly perfused tissues, mainly CNS, eyes, skeletal muscle, and subcutaneous tissue, in this order [42–46].

Neurocysticercosis is the most frequent parasitic disease of the CNS. It can be subarachnoid-cisternal (the most frequent form), parenchymal (the second most common and typically located in the corticomedullary junction), intraventricular, and spinal [53, 56–58]. It can be asymptomatic or have symptoms depending on the location, number, size, and stage of the lesions, and the aggressiveness of the immune reaction. The most frequent clinical presentation is epilepsy, followed by headache and symptoms derived from cerebrospinal fluid (CSF) obstruction. Neurocysticercosis is the most common cause of acquired epilepsy in developing countries [55, 59].

### Imaging findings

As neurocysticercosis is the most common form of this parasitic infection this chapter concentrates in its imaging findings. Neurocysticercosis is classified in five stages regarding their evolution from active larvae to dead lesions due to natural degeneration, host immune response and/or therapy: non-cystic, vesicular, colloidal vesicular, granular nodular, and calcified nodular (Table 3). This degeneration can last years, and a combination of the different stages can be seen in the same individual [60, 61].

**Table 3:**
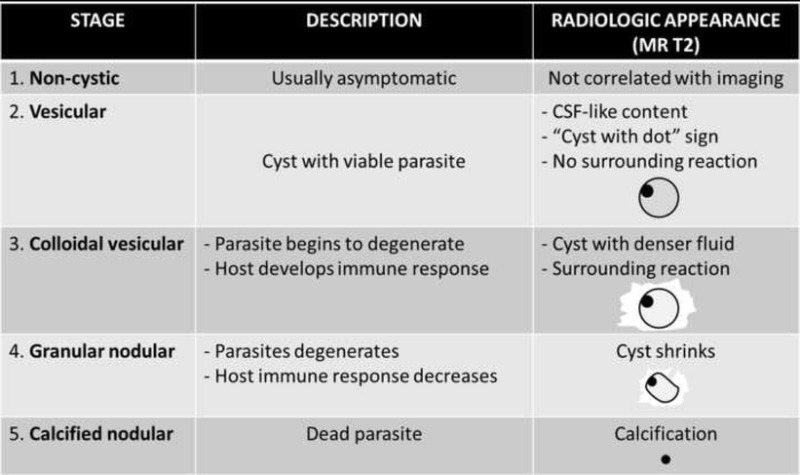
Main stages of neurocysticercosis in imaging

**Fig. 8:**
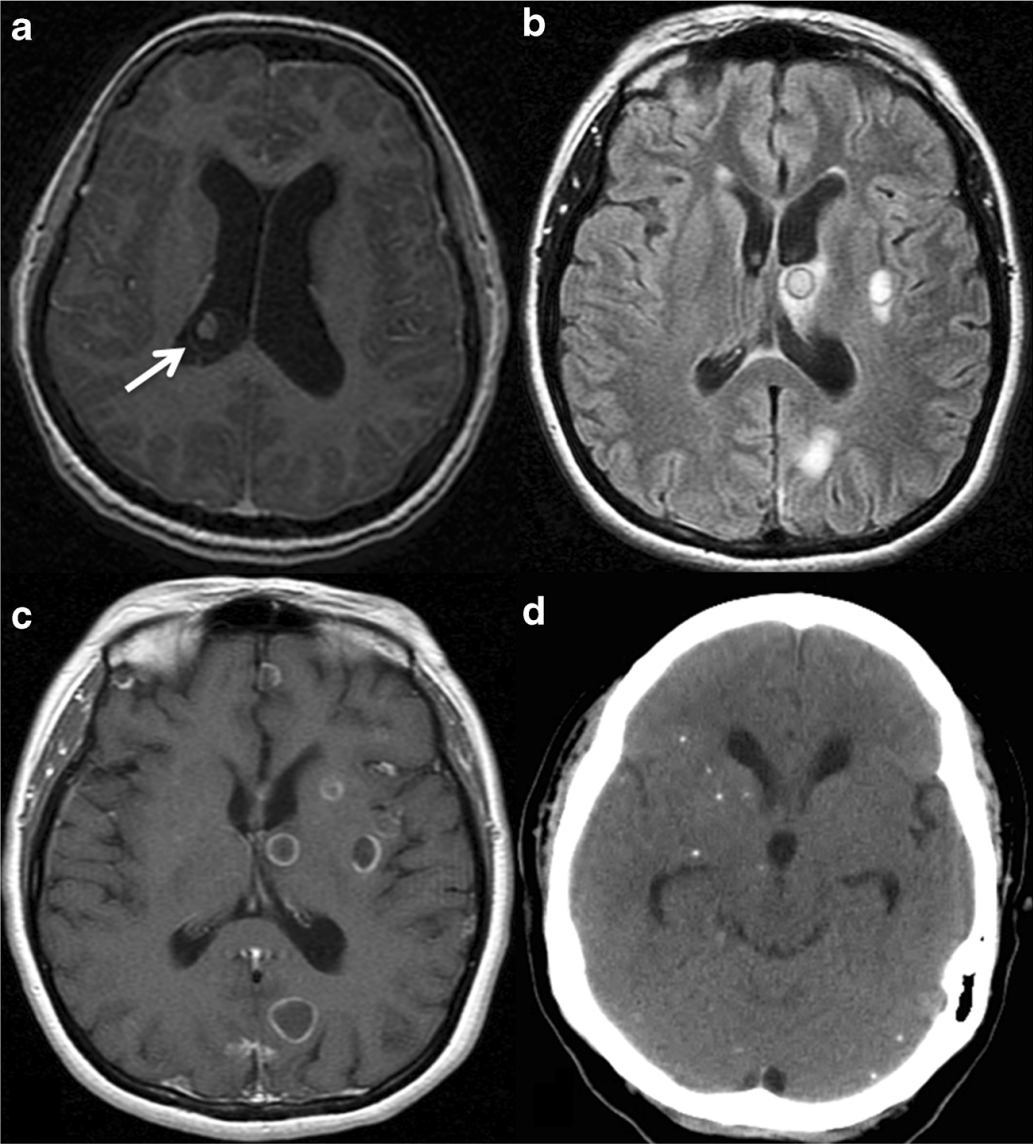
Neurocysticercosis. **a** Vesicular stage. Gadolinium-enhanced axial T1-weighted MR image. Cystic lesion in the right ventricular atrium (*arrow*) whose content similar to the CSF; inside there is an eccentric dot with high signal intensity representing the protoscolex (“cyst with dot” sign). There is no enhancement as there is no or scant host reaction in this stage. **b** Colloidal vesicular stage. Axial fluid-attenuated inversion recovery MR image. There are several lesions located in the subarachnoid space and corticomedullary junction and one in the left thalamus, they have a content, which is denser than the CSF (is has a higher signal intensity) and are surrounded by marked oedema. **c** Colloidal vesicular stage. Gadolinium-enhanced axial T1-weighted MR image of the same patient as in **c**. The lesions show ring enhancement. **d** Calcified nodular phase. CT image showing multiple calcified lesions dispersed in the brain parenchyma mostly in the corticomedullary junction and subarachnoid space, and some of them in the right basal ganglia. They represent calcified granulomata in dead neurocysticercosis

Non-cystic neurocysticercosis is the first stage, usually asymptomatic and not correlated with imaging findings. The vesicular stage is a cyst or a group of cysts (racemose form) containing the larvae or protoscolex. The cyst has content similar to the CSF in CT and MR and the protoscolex can be seen inside as an eccentric dot with high signal intensity on T1WI, the “cyst with dot” sign. As there is no or scant host reaction, little or no surrounding oedema, and no enhancement is seen (Fig. 8a) [55, 60, 62, 63]. In colloidal vesicular stage the parasite begins to degenerate and the host develops a stronger immune response. The fluid of the cyst turns denser and thus appears with higher signal on MR and higher attenuation on CT. The inflammatory response leads to surrounding oedema and ring enhancement of the cyst (Fig 8b-c) [55, 64]. In the granular nodular phase the cyst shrinks, the acute immune response decreases, and it is progressively replaced by gliosis, imaging findings can be similar to colloidal vesicular stage. The end stage, the calcified nodular phase, represents the dead parasite. CT is superior to MR when detecting the calcified lesions of this stage (Fig. 8d) [55].

**Fig. 9:**
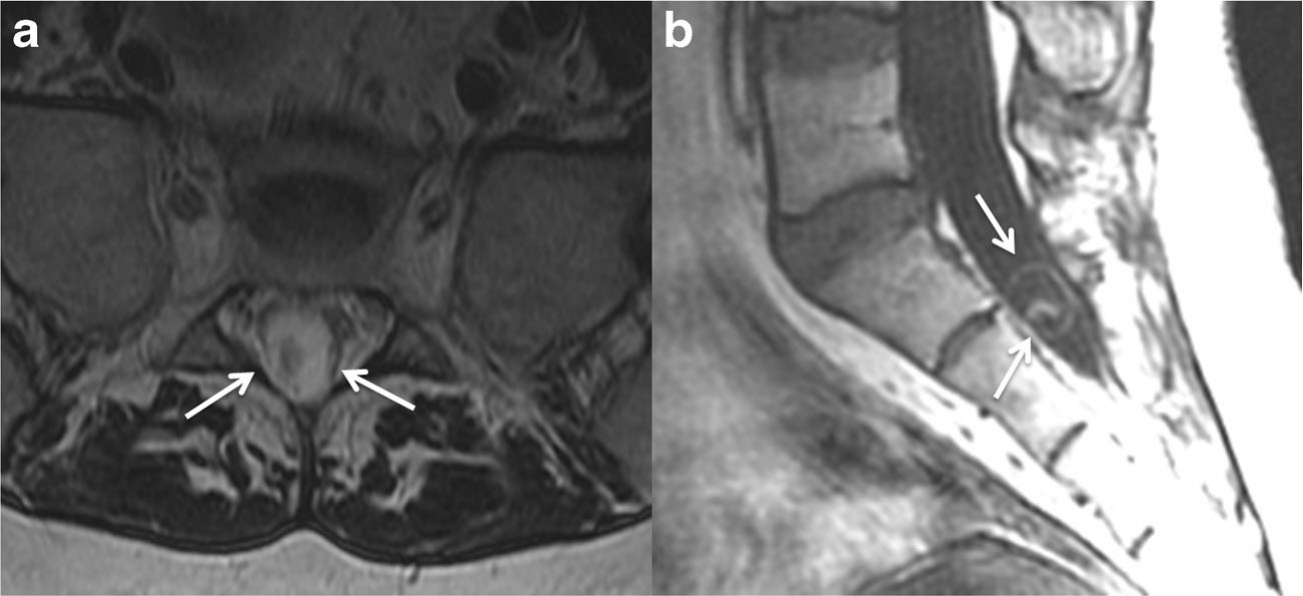
Spinal neurocysticercosis. **a** Axial T2-weighted MR image, showing a cystic lesion in the subarachnoid space of the dural sac in the sacrum. There is a dot with lower signal intensity inside the cyst representing the protoscolex (“cyst with dot” sign) in a vesicular stage. **b** Coronal T2-weighted MR image of the same patient as in **a**. The intradural cyst (*arrows*) has a signal intensity similar to the CSF, note also the thin walls and the high signal intensity protoscolex inside the cyst, these are typical findings of a vesicular stage neurocysticercosis

Intraventricular and spinal cysticercosis rare and usually caused by CSF seeding Figs. 8a and 9.

Patients can also develop other complications such as arteritis and arachnoiditis.

### Diagnosis and treatment

Diagnosis of neurocysticercosis is based on a combination of clinical examination, medical records, imaging findings (CT and MR) and a serological or CSF detection of antibodies. Sometimes radiologists are the first ones to find cysticercosis lesions and thus play an important role in detecting or raising the suspicion of this parasitic disease.

Treatment of active neurocysticercosis is based on antiparasitic drugs (mainly praziquantel and/or albendazole) sometimes combined with steroids. Intraventricular or spinal cysts may require surgical removal.

## Clonorchiasis

### The parasite, its cycle, and human infection

Clonorchis sinensis is a liver fluke, endemic along river basins in East Asia, including Northeast China, Manchuria region, Korea, Amur basin in Russia, Taiwan, and Vietnam [65]. Opisthorchis viverrini and O. felineus have a very similar life cycles and almost indistinguishable clinical and imaging findings [66].

 C. sinensis life cycle requires 3 different hosts: fresh water snails (Parafossarulus spp and Bithynia spp mainly), fresh water fish (Cyprinid family), and mammals (e.g. humans, dogs, cats, pigs, rats, badges, or weasels). The snails eat C. sinensis eggs, which will turn into the larval stage (cercariae). Cercariae are released to water and actively invade the mucosae or skin of the fish becoming encysted (metacercariae) in their muscles and soft tissues. [67, 68].

Definitive hosts, including humans, are affected when eating raw or poorly cooked infected fish. Metacercariae are liberated in the duodenum and migrate to the biliary tree through the ampulla of Vater, lodging in the small intrahepatic bile ducts where they turn into the adult worm. Released eggs go downstream into the duodenum again and are discharged with the host faeces closing the life cycle [67, 68].

 C. sinensis is well adapted to human bile ducts and is able to colonise them with few or no symptoms at all. Most of the clinical manifestations and, therefore, imaging findings, depend on the amount of flukes inside the biliary tree and are due to bile duct obstruction leading to cholangitis (oriental cholangio-hepatitis).

Major complications are gallstones, stones, and casts inside dilated bile ducts, pyogenic cholangitis, liver abscesses, cholecystitis, hepatitis, or even cirrhosis. Pancreatic ducts can be affected in heavy infections [62–66].

 C. sinensis is a known risk factor for cholangiocarcinoma due to chronic inflammation and damage of the biliary epithelium [63, 68–71]. Some authors have also proposed additional potential associations of clonorchis with other biliopancreatic neoplasms, for instance, pancreatic mucinous cystadenoma [72], although probably further investigations are needed.

### Imaging findings

Typical imaging findings are diffuse dilatation of peripheral intrahepatic bile ducts where the flukes are lodged. Central intrahepatic and extrahepatic ducts are often spared.

**Fig. 10:**
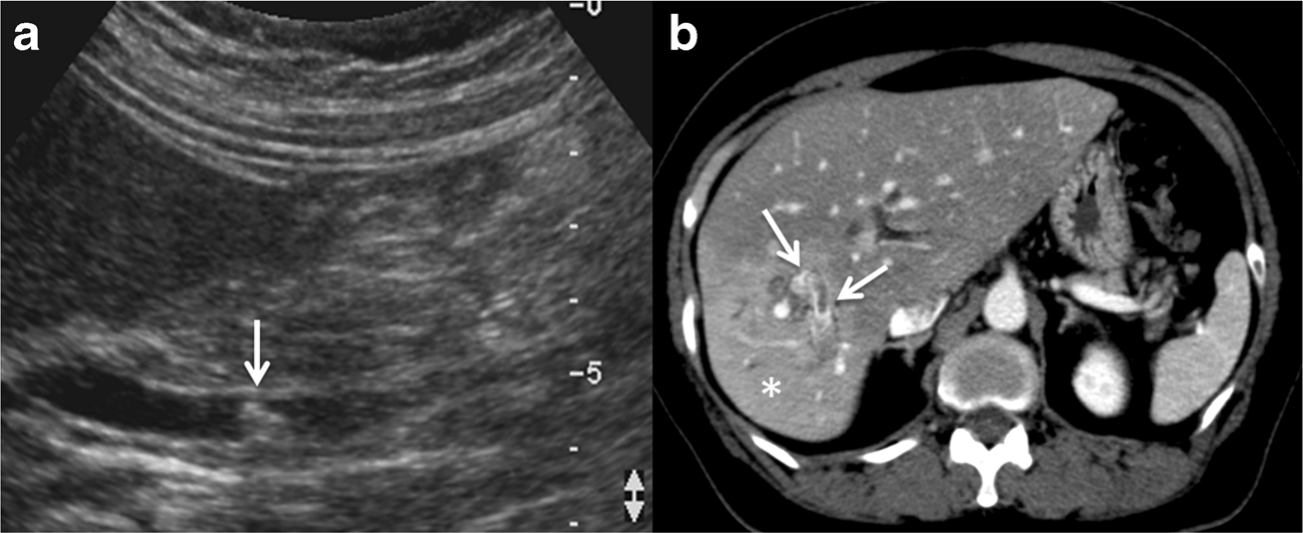
Clonorchiasis in a 50-year-old woman native to China. **a.** Axial US image shows mild choledocal dilatation with a non-obstructive bile stone. **b.** Contrast-enhanced CT image shows dilatation of intrahepatic bile ducts in the right lobe, with periductal enhancement and intraluminal solid contents (debris, parasites), including bile stones and casts (*arrows*). Note the hyperaemia of the surrounding hepatic parenchyma (*asterisk*), secondary to acute inflammatory changes

US is used as screening technique in endemic areas. It characteristically displays the dilated intrahepatic ducts with wall thickening and a surrounding hyperechoic halo thought to be caused by periductal fibrosis. The gallbladder and bile ducts can be filled with detritus and/or gallstones, and sometimes the flukes themselves can be seen as moving echoic foci (Fig. 10a).

CT and MR, especially cholangiographic sequences, also show the dilated bile ducts with thickened walls. Periductal enhancement after iv contrast injection indicates active inflammation (Fig. 10b) [68, 72–75]. MR cholangiography can show the flukes as low-signal material within the dilated ducts. Cholangiogram has a high sensitivity in detecting the parasites, which appear as linear or ovoid filling defects up to 10 mm [69, 76–78].

Clonorchiasis can mimic other chronic inflammatory biliary processes, namely recurrent pyogenic cholangitis and primary sclerosing cholangitis, forcing the radiologist to be precise and correlate closely with the clinical picture and history of the patient [77].

### Diagnosis and treatment

Diagnosis is made by demonstration of the eggs in the host’s faeces, biliary, or duodenal aspirates. Also, DNA of the fluke can be detected in the faeces and serum antigens can be identified with ELISA tests [67, 79]. Praziquantel is the treatment of choice.

## Schistosomiasis

### The parasite and its cycle

Schistosomiasis is caused by six species of Schistosoma flukes, which have the human body as definitive host. There are two major forms of schistosomiasis: intestinal and genitourinary, but all Schistosoma species have a similar life cycle.

Schistosoma eggs are released with faeces or urine (depending if it is an intestinal or genitourinary infection). When the eggs are discharged in water they liberate the larvae (miracidium) which infect the intermediate host (some species of aquatic snails) where they mature to secondary larvae (cercariae). Cercariae leave the snails and swim to invade their final host, the human being. They penetrate through the skin and enter blood and lymphatic vessels where they become schistosomulae and lodge finally in the liver sinusoids turning into male–female pairs to copulate. Adult worms migrate downstream to the mesenteric and rectal venules (intestinal schistosomiasis) or to the pelvic and paravesical venous plexus (genitourinary schistosomiasis), where they live and produce eggs [80]. Eggs migrate through the bowel o urinary bladder wall and are discharged with the stools or urine closing the cycle.

## Intestinal schistosomiasis

### Human infection

The main causes are S. mansoni (Central Africa, Middle East, the Caribbean, and South America) and S. japonicum (China, the Philippines, and South East Asia). The rest of species have very limited incidence. Infection is more prevalent and intense in children and most of the cases (90 %) take place in Africa [80].

Clinical picture depends on the stage of the disease. When cercariae penetrate the skin they can cause dermatitis (“swimmer’s itch” or “lake itch”). The migration and maturation of the schistosomulae may produce a systemic inflammatory reaction (“Katayama fever”) with flu-like symptoms, hepatosplenomegaly, and eosinophilia. When adult worms lodge in the mesenteric venules they can cause colitis and iron-deficiency anaemia. Symptomatology can be subtle though, and individuals can remain nearly asymptomatic [80]. Some schistosoma eggs are trapped in portal venules causing a granulomatous reaction and periportal hepatic fibrosis with relative sparing of liver acinar architecture in contrast with viral cirrhosis. [81–83].

It has been suggested that the chronic hepatic injury caused by schistosomiasis may enhance the liver damage in patients with chronic viral hepatitis and, therefore, be a potential cofactor for the development of hepatocellular carcinoma [84].

### Imaging findings

Most of the imaging findings are actually due to the chronic hepatic affection and its related complications such as portal hypertension.

**Fig. 11:**
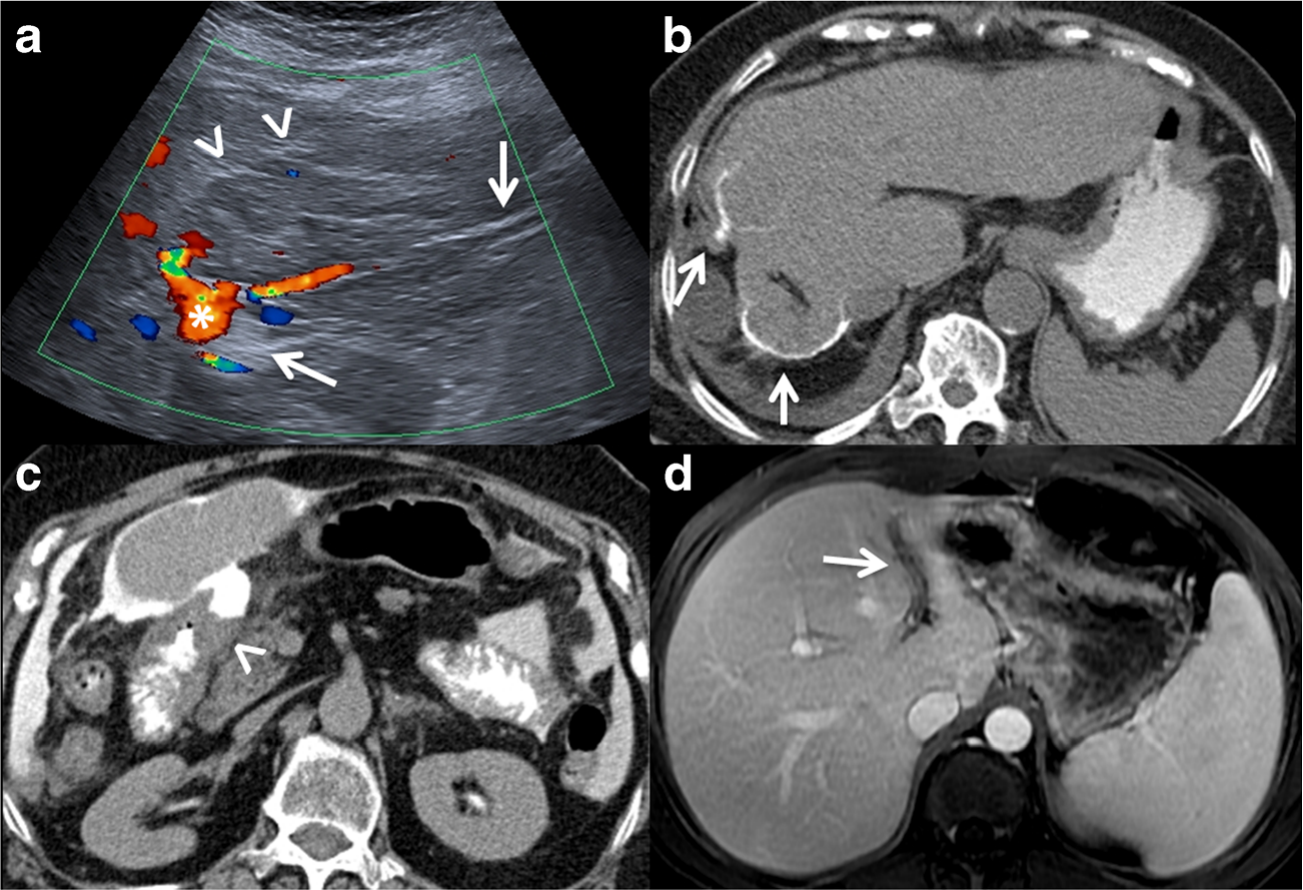
Intestinal schistosomiasis. **a.** US-Doppler axial image from a Filipino patient with chronic infection by S. japonicum. Note also the stigmata of chronic liver disease (caudate and left lobes hypertrophy, heterogeneous parenchyma and irregular surface). Periportal fibrosis appears as echogenic cuffing around portal vessels (*arrows*), note left portal vein (*asterisk*). Characteristic fibrotic septa are seen perpendicular to the surface (*arrowheads*). **b.** Unenhanced CT image with iodinated oral contrast from the same patient as in figure a. It shows fibrosis with calcified septa (“turtle back” appearance) in the right liver lobe (*arrows*). Note also the imaging findings consistent with chronic liver disease (atrophy of the right liver lobe with hypertrophy of the left and caudate lobes). **c.** A month later the patient from figures a and b came to the ER with acute abdominal pain; unenhanced CT with iodinated oral contrast was performed, showing a perforated duodenal ulcer with oral contrast leakage (*arrowhead*), microbiological and pathological exams probed that it was a duodenal ulcer caused by S. japonicum. **d.** Gadolinium-enhanced axial T1-weighted MR image with fat suppression of a patient from Guinea-Conakry with chronic S. mansoni infection, it shows almost complete atrophy of the left portal vein and periportal fibrosis, seen as a hypointense periportal cuffing (*arrow*)

US can display periportal fibrosis as echogenic cuffing around the portal branches. The presence of peripheral hepatic vessels is also characteristic. US can also reflect the typical imaging findings shared with other chronic hepatopathies such as irregular liver surface, heterogeneous parenchyma, or caudate and left lobe hypertrophy with right atrophy (Fig. 11a). Portal hypertension if present can manifest with dilatation of the portal-splenic-mesenteric axis, diminished portal velocity or even hepatofugal flow, or splenomegaly [85–88].

 S. japonicum chronic hepatic infection has a characteristic pattern of macroscopic fibrotic septa that reach liver surface and appear in the US as hyperechoic bands sometimes calcified (“mosaic-like” pattern; Fig. 11a) [87].

CT can also display the typical findings of a chronic hepatopathy and portal hypertension. Periportal fibrosis appears as hypoattenuating bands around portal branches that can enhance after iv infusion of iodinated contrast. The characteristic pattern of septal fibrosis seen in S. japonicum shows partially calcified hepatic septa that reach perpendicularly the liver capsule (“turtle back” appearance; Fig. 11b-c) [85, 89].

MR imaging of chronic hepatic schistosomiasis shows periportal cuffing with high signal on T2WI and iso to hypointense on T1WI Fig. 11d. MR can also show fat hypertrophy around the hepatic hilum and gallbladder fossa with intrahepatic extension of the fat. Chronic hepatopathy and portal hypertension features can also be seen [82, 90, 91]. The fibrotic septa of S. japonicum are hypointense on T1WI and hyperintense on T2WI [4].

Acute schistosomiasis is rarely seen in imaging. Some authors have described hepatomegaly with multiple nodules, hypoattenuating on CT, hypointense on T1WI, and hyperintense on T2WI, apparently corresponding to granulomata [92, 93].

## Genitourinary schistosomiasis

### Human infection

Caused by S. haematobium, which is endemic in areas of sub-Saharan Africa, Egypt, and the Middle East. In recent years some cases have been reported in Corsica, and intermediate snail hosts have also been found in other Mediterranean regions of Europe although non-infected with schistosoma. This poses a potential risk of future expansion of urinary schistosomiasis [94, 95].

Eggs trigger a granulomatous inflammatory reaction in the bladder and distal ureter walls, causing urothelial proliferation and metaplasia (cystitis cystica, ureteritis cystica, and cystitis glandularis), which eventually ends in fibrosis and calcification that can cause urinary obstruction [96].

Typical clinical manifestations are haematuria and cystitis-like symptoms. Urinary lithiasis and bacterial superinfection are potential complications.

### Imaging findings

**Fig. 12:**
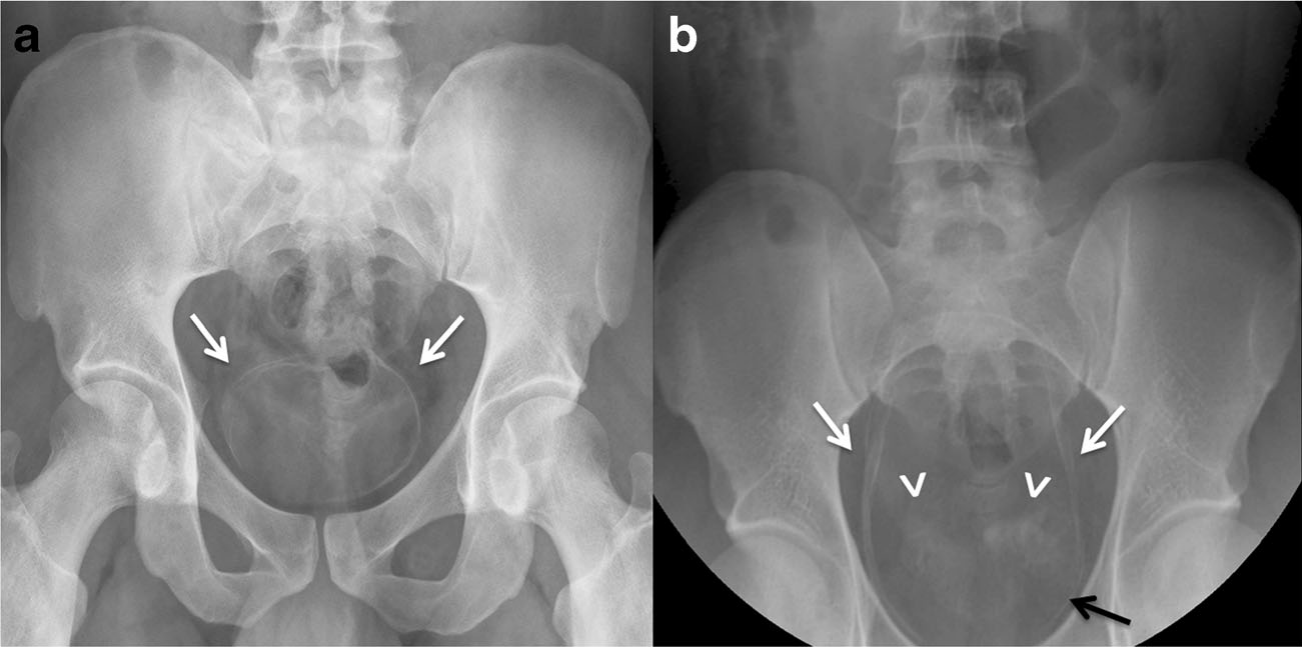
Genitourinary schitosomiasis. **a.** Pelvis radiograph of a patient born in Ivory Coast, showing a complete calcification of the urinary bladder walls (*arrows*), pathognomonic of chronic urinary schistosomiasis. **b.** Pelvis radiograph from an iv urography in a patient born in Mali; there is wall calcification of both distal ureters (*white arrows*) and anterior urinary bladder wall (*black arrow*), and also calcification of the seminal vesicles (*arrowheads*), consistent with chronic schistosomiasis. Cystoscopy was performed in both patients revealing characteristic urothelial lesions. Biopsies showed schistosoma eggs

Plain radiographs or CT can demonstrate linear calcifications mainly in the walls of the bladder and distal ureters. A totally calcified bladder resembling a foetus head in the pelvis is pathognomonic of chronic urinary schistosomiasis (Fig. 12a). The degree of calcification correlates with the number of eggs in the tissue.

Less frequently, other genitourinary structures such as the urethra, prostate, seminal vesicles, vasa deferentia, or even vagina and testicles, can also be affected by egg deposition (Fig. 12b).

CT detects smaller and earlier calcifications. Cystitis cystica, ureteritis cystica, and cystitis glandularis appear as intraluminal polypoid filling defects inside the urinary tract. Vesicoureteral reflux and urinary obstruction can appear in long-standing cases [96, 97].

US can show earlier changes in the bladder wall, such as irregular wall thickening and polypoid lesions that can mimic carcinoma [80].

Urinary schistosomiasis is a risk factor for squamous cell carcinoma due to the chronic harm in the urothelium and squamous metaplasia. In endemic areas squamous carcinoma represents more than 50 % of bladder malignancies [98].

### Diagnosis and treatment

Diagnosis is made by detection of the eggs in faeces, urine, or affected tissues. Antigens and antibodies in serum or urine can help staging the infection. DNA of the fluke can be found in sera and faeces with PCR [80]. Treatment is praziquantel.

## Fascioliasis

### The parasite, its cycle, and human infection

It is caused by two species of Fasciola fluke, F. hepatica, and F. gigantica. Fasciolaliasis is a major veterinary and health problem in developing areas of the Andean region (mainly Bolivia, Peru, and Ecuador), the Caribbean (Cuba and Puerto Rico), Northern Africa (principally Egypt), and Iran and the Caspian Sea region. There are also areas of higher incidence of fascioliasis in Europe, including Western Europe (Portugal, France, Spain, and Britain), Turkey and the former USSR [79, 99].

Adult flukes live in the biliary tract of its definitive hosts (herbivorous mammals such as cattle, sheep, and goats) and need an intermediate host to complete its life cycle (some species of freshwater snails). Humans can be definitive hosts.

Eggs are released with the definitive host faeces. In contact with water they liberate miracidiae that infect the snails. Snails release cercariae that remain encysted (metacercariae) in aquatic plants (e.g. watercress, corn salad, algae, or mint). Humans become infected by eating vegetables with metacercariae or freshwater containing cercariae. Metacercariae release juvenile flukes in the duodenum, which migrate towards the liver crossing the duodenal wall and the peritoneal cavity. Once in the liver the flukes move through the parenchyma searching the large intrahepatic bile ducts which are the permanent residence of the adult forms.

Liver infection (hepatic phase) typically causes hepatitis-like symptoms, urticarial and eosinophilia. Eggs are released with the bile and are expulsed with the stool closing the cycle. Biliary tree infection (ductal phase) can cause right upper quadrant pain and cholestasis. Possible complications are hepatic subcapsular haemorrhage due to the entry of the flukes, cholangitis, hepatic abscesses, or cholecystitis, either caused by the flukes and/or bacterial superinfection [100].

### Imaging findings

The key to understanding the imaging findings is to remember the migration route of this worm.

The hepatic phase on US can range from small, subcapsular hypoechoic lesions of ill-defined borders and tendency to converge, to more diffuse areas of parenchymal affection and heterogeneous echogenicity that can mimic malignancy. These lesions are the tracts of the migrating larvae. The ductal phase can show dilatation of bile ducts with wall thickening and sometimes tortuous shape. Solid contents in the ductal lumina or inside the gallbladder can correspond to debris or the flukes themselves [7, 100–102].

**Fig. 13:**
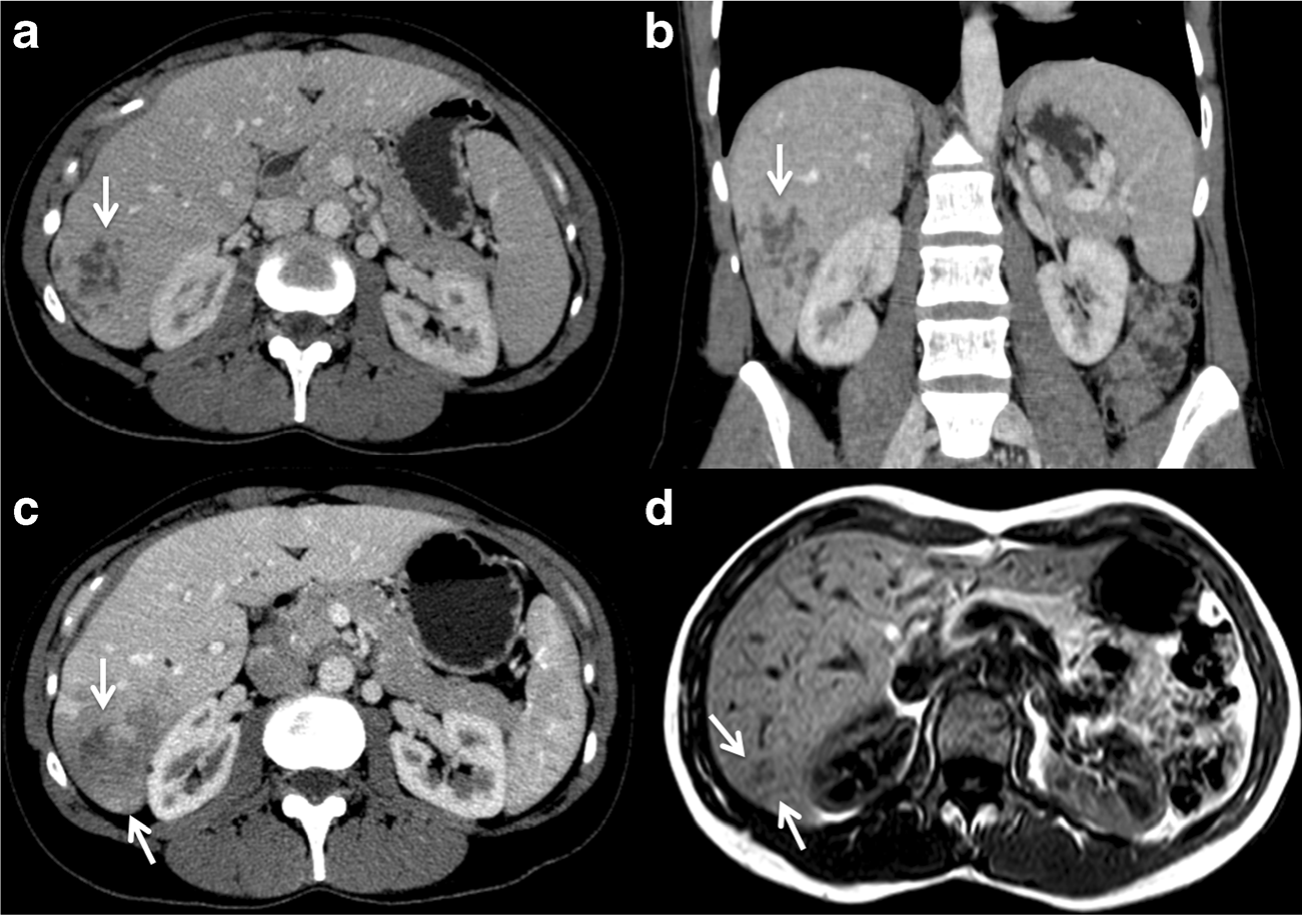
Fascioliasis. 42-year-old woman with history of long-term fever. **a-b** Axial and coronal images of contrast enhanced-CT shows multiple subcapsular and confluent lesions in right hepatic lobe (*arrows*). Some of the lesions have a tubular shape. **c.** Contrast enhanced-CT image of the same patient 3 weeks later; the lesions have converged forming a greater ill-defined lesion with central areas compatible with necrosis/abscess (*arrows*). The patient presented with eosinophilia and positive serology for fasciola and was treated with antiparasitic drugs. **d.** Axial T1-weighted MR image without contrast a month later, demonstrating improvement of the hepatic affection, only residual tubular and pseudonodular lesions of low signal intensity remain (*arrows*)

CT can show the characteristic subcapsular liver lesions of the hepatic phase as hypoattenuating small round nodules with rim enhancement that tend to form clusters or tract-like lesions (Fig. 13a–b). Parenchymal ill-defined areas of low-attenuation have also been described (Fig. 13c). The ductal phase can demonstrate biliary dilatation, duct wall enhancement, and periportal oedema. Calcifications are rare. Hepatic abscesses characteristically have a thick hypoattenuating rim with poor enhancement, do not tend to merge and evolve slowly, in contrast to pyogenic or amoebic abscesses [100, 101, 103].

MR can show hepatic capsular thickening with high signal on T2WI and/or capsular enhancement. The migration paths and lesions caused by larvae in the liver appear as subcapsular lines and nodules that tend to cluster, with high signal intensity on T2WI and low signal on T1WI (Fig. 13d). These lesions can have rim-enhancement after iv contrast administration. Biliary ducts dilatation is better depicted on cholangiographic sequences. Sometimes adult worms can be seen as low-signal-filling defects inside the central or extrahepatic bile ducts [7, 100, 104].

ERCP can also display the adult flukes as curvilinear filling defects in the biliary ducts [4].

The presence of a subcapsular hepatic haematoma with eosinophilia is highly suspicious of either hepatic fascioliasis or polyarteritis nodosa [100].

### Diagnosis and treatment

Diagnosis is confirmed by finding the fluke or eggs in the faeces, duodenal fluid, or biopsy/surgically-obtained tissue. Also, with the detection of antibodies in plasma or antigens in serum or stool [100]. Triclabendazol is the drug of choice [68].

Radiologists can drain bigger liver lesions with US or CT guidance. Also, diagnostic imaging tests are useful tools to evaluate the response to the treatment.

## Ascariasis

### The parasite, its cycle, and human infection

 Ascaris lumbricoides is the most common helminthic infestation affecting the human. Ascariasis strikes millions of people worldwide, especially in tropical and subtropical areas of developing countries in Africa, Latin America, and Asia. Children are at special risk of developing more severe infestations [68, 105, 106].

Humans are the final host. Infection starts after the ingestion of eggs, usually in food, water, or soil contaminated with a carrier’s faeces. Eggs hatch in the small bowel and the larvae perforate the wall entering the portal system or lymphatics ending up in the lung, where they penetrate into the alveoli.

Pulmonary ascariasis can manifest with pneumonia or bronchitis-like symptoms. Patients can sometimes develop Löffler syndrome, a type of secondary eosinophilic lung disease.

The larvae migrate to the airway and travel up to the larynx, where they are swallowed and enter the digestive tract. Worms mature in the small bowel lumen, mainly jejunum, and ileum, reaching up to 35 cm length. After copulation, eggs are released with stools [105, 107].

The adult worms living in the bowel can cause none or non-specific symptoms such as abdominal pain or discomfort, nausea/vomiting, or diarrhoea. Big amounts of worms can cause intestinal obstruction, especially in children [107].

Seldomly, worms can enter the biliary tree or pancreatic duct and can cause cholangitis, cholecystitis, hepatic abscesses, or pancreatitis. Usually biliary infestation affects the extrahepatic bile ducts. Repeated infections can lead to recurrent pyogenic cholangitis [68, 107].

### Imaging findings

Barium fluoroscopy studies and US continue to play a major role in diagnosing and evaluating intestinal and biliary ascariasis, especially in the developing world.

**Fig. 14:**
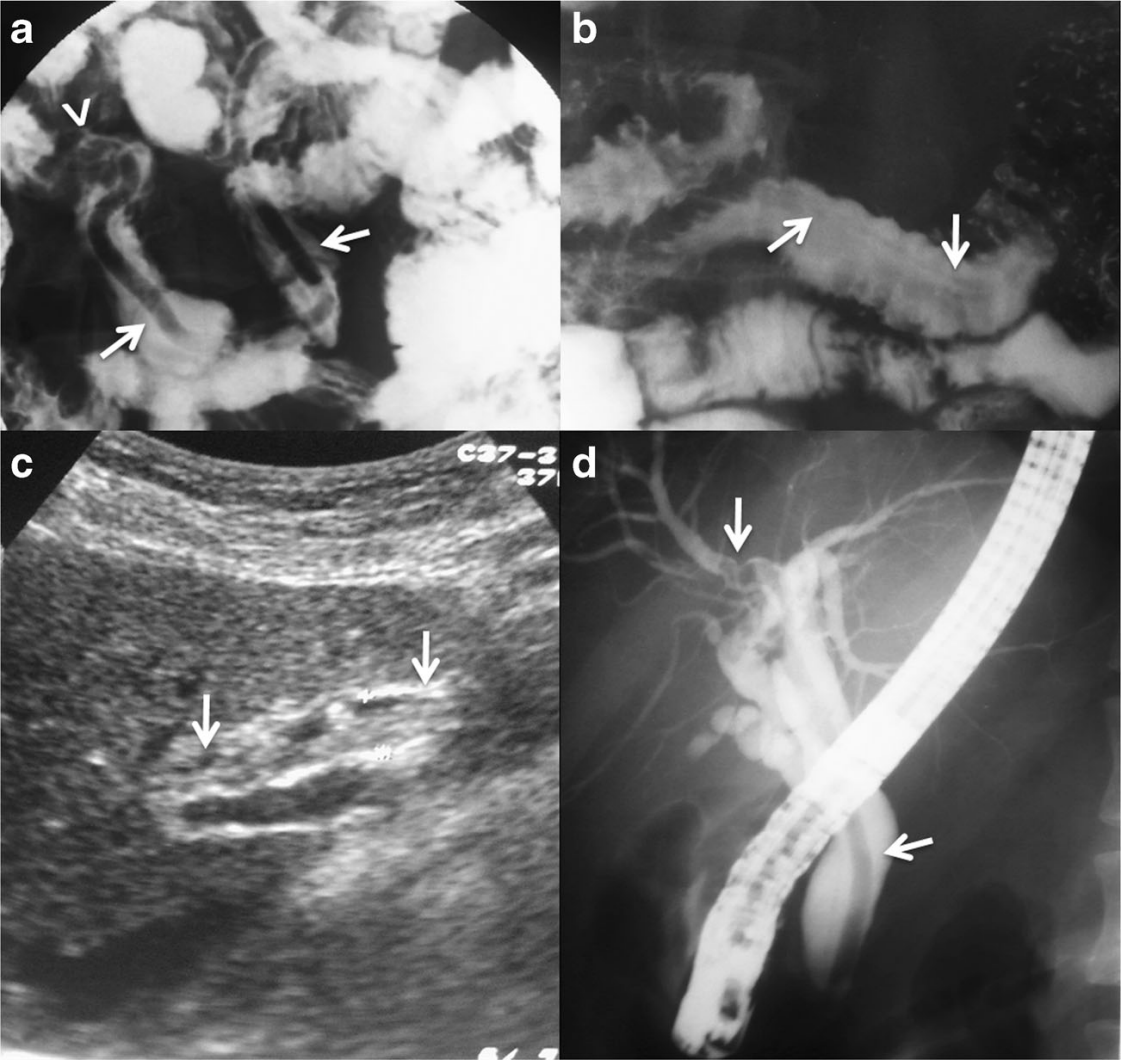
Ascariasis. **a.** Barium fluoroscopic study of a 24-year-old woman from Ecuador with previous history of ascariasis and presenting with abdominal pain, diarrhoea, and anaemia. The study shows a worm inside a jejunal loop (*arrows*). The faeces examination revealed ascaris eggs and the patient was successfully treated with mebendazole. Note that the head (blunt) of the worm points proximally (*arrowhead*), as is usual in this parasite **b**. Barium fluoroscopic study of a 37-year-old male from Ecuador presenting with intermittent episodes of right lower quadrant pain, mild vomiting, diarrhoea, and fever. Stool parasitic study revealed ascaris eggs. The barium examination shows intestinal worms compatible with ascaris, note that the shown worm has swallowed barium contrast (*arrows*). **c **and** d.** 29-year-old woman from Ecuador with previous surgery of cholecystectomy, presenting with biliary vomiting, right upper quadrant pain, and elevated liver and cholestatic enzymes. The US examination in C displayed a long echogenic filling defect without acoustic shadowing inside the common bile duct (CBD) (*arrows*), with other adjacent smaller filling defects compatible with gallstones and/or debris. The ERCP in B shows a worm inside the common bile duct whose head is introduced in the right hepatic duct (*arrows*). The living worm was extracted with ERCP and proved to be an ascaris lumbricoides

Barium examination reveals the adult worms in the bowel lumen as tubular smooth radiolucent filling defects. The head of the worm is blunt and the rear pointed, and most of them are seen with the head pointing proximally (Fig. 14a). Sometimes they can be seen moving, and if both the head and tail point distally the worm is likely to be dead or stunned by treatment. If the patient has been fasting the ascaris may ingest the barium and appear filled with the oral contrast (Fig. 14b) [107].

Alteration of the bowel wall secondary to the infestation can be sometimes visible as slight thickened mucosal folds [107].

US shows the adult worms as long echogenic filling defects without acoustic shadowing inside the intestinal lumen. Higher resolution linear probes can reveal more detailed anatomy of the worm. Also, as in barium studies, worms can be seen moving in real time. Worms inside the biliary tree or pancreatic duct are seen as inside the bowel. Gallbladder or biliary tree can show wall enlargement and intraluminal debris related to the parasitic infection (Fig. 14c) [105, 107, 108].

CT is rarely used as a diagnostic tool but ascaris can be unexpectedly found as elongated filling defects in the lumen of the intestine [105].

MR shows the worms as T2 hypointense and T1 iso to hypointense cylindrical filling defects inside the bowel lumen or biliary tree. MR cholagiopancreatographic sequences can also display the worms as hypointense tubular structures [105].

Endoscopic retrograde cholangiopancreatography (ERCP) shows ascaris also as tubular filling defects and can be used to remove the worms (Fig. 14d).

Pulmonary ascariasis typically manifest in chest radiographs and CT as patchy “ground glass” or alveolar infiltrates that usually resolve within 10 days [18, 105].

### Diagnosis and treatment

Direct identification of the worms in stool or microscopic identification of the eggs in faeces. Eggs can also be identified in other fluids such as vomitus, sputum, or bowel aspirate [105].

Albendazole and mebendazole are the drugs of choice [106].

## Strongyloidiasis

### The parasite, its cycle, and human infection

 Strongyloides stercolaris is a worm that inhabits the small bowel of human hosts. It occurs in the tropics and has also been reported in more temperate climates (e.g. southern parts of North America, southern Europe, and Britain) [109].

Female parasites lay eggs in the small intestinal mucosa that soon release microscopic larvae, which usually escape at the non-infective stage (rhabditiform) in the faeces and develop into free-living adult worms within a week. The free-living females produce another generation of rhabditiform larvae, which develop into infective filariform larvae. Humans are infected by penetration through intact skin. S. stercolaris is the only soil-transmitted helminth infecting humans in which the worm can multiply in the free-living stage. After penetrating the skin, the larvae are carried to the lungs, and migrate through the alveoli to reach the bronchi. They migrate upstream the bronchial tree and are swallowed to reach their normal habitat in the small bowel [109, 110].

The unique ability of this nematode to replicate in the human host permits cycles of autoinfection (they may reinfect the same host by either penetrating the perianal skin or the bowel wall) leading to chronic disease that can last for several decades eventually resulting in massive parasitic infestation. As ascaris lumbricoides, Strongyloides stercolaris can also trigger an eosinophilic pneumonia (Löffler’s syndrome) [109–114].

Many infections are asymptomatic. Acute infection may be associated with coughing and wheezing, abdominal pain, and diarrhoea [109, 114]. In chronic strongyloidiasis intestinal symptoms may be present, which are usually vague (irregular bouts of looseness of the stools). Larva currens (“creeping eruption”) is a characteristic, virtually pathognomonic, skin eruption caused by the migration of larvae through the skin during autoinfection [109].

Hyperinfection syndrome is a rare complication that occurs when host immunity is significantly and usually abruptly reduced, allowing rapid and disseminated migration of filariform larvae into tissues. It includes severe bloody diarrhoea, bowel inflammation with microperforations, bacterial peritonitis, septicaemia, pulmonary exudates, haemoptysis, pleural effusion and hypoxia, encephalitis, and even bacterial meningitis [109–116].

### Imaging findings

**Fig. 15:**
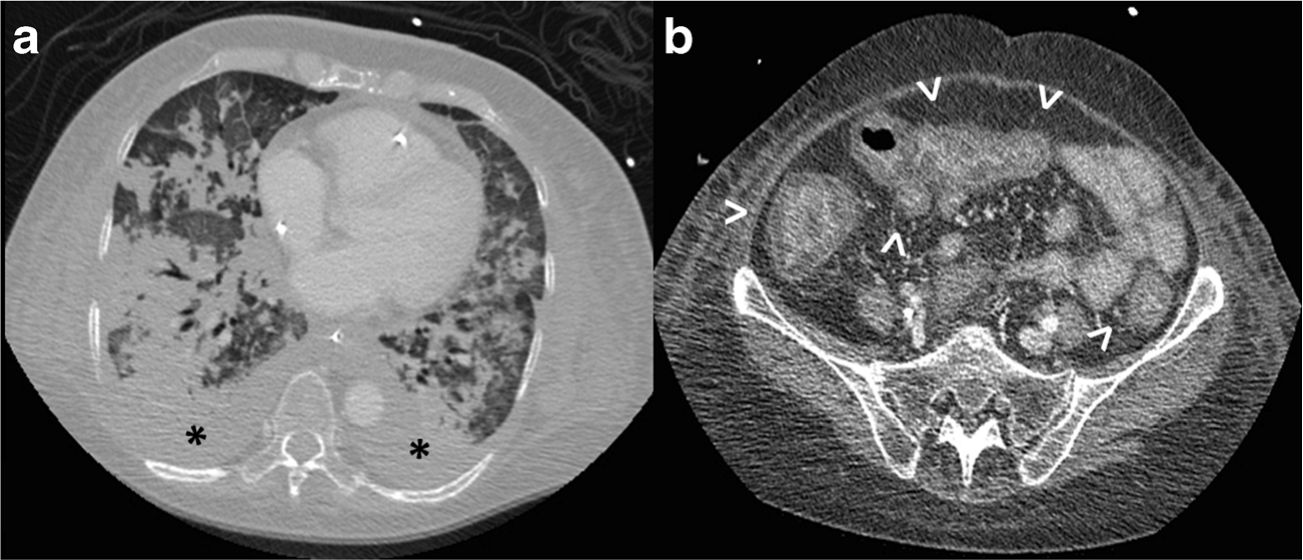
Strongyloidiasis. **a.** Chest CT image of an inmunocompromised 45-year-old patient presenting with a severe hyperinfection syndrome. The scan shows bilateral widespread pulmonary infiltrates and pleural effusion (*asterisks*). **b.** Contrast-enhanced CT image from the same patient as in **a**. The patient’s condition worsened after a week and the CT scan showed wall oedema in several bowel loops (*arrowheads*). Strongyolides larvae were found in the faeces and in bronchial aspirate

Most patients with pulmonary symptoms of strongyloidiasis have abnormal findings on chest radiographs [114, 117, 118]. During the phase of autoinfection, chest radiographs or CT may show fine miliary nodules or diffuse reticular interstitial opacities. With the development of heavier infection, bronchopneumonia with scattered, patchy alveolar opacities, segmental opacities, even lobar migratory opacities may be present [114, 117]. In patients with hyperinfection syndrome the massive migration of larvae through the lungs typically produces extensive pneumonia, pulmonary haemorrhage, and pleural effusion (Fig. 15a). Pulmonary cavitation and abscesses can occur usually due to secondary bacterial infection [114, 115].

When malabsorption is present the radiographic findings are similar to those of tropical sprue, including increased diameter of the small bowel lumen, generalized hypotonia, and wall oedema (Fig. 15b) [110, 112, 114].

### Diagnosis and treatment

Diagnosis of strongyloidiasis is notoriously difficult. Direct stool microscopy, stool culture, duodenal biopsy, and serological tests (e.g. ELISA) may be useful. However, despite all these techniques, diagnosis may remain in doubt and clinical features should remain part of the diagnostic process. High eosinophilia, unexplained diarrhoea and a typical larva currens rash are all highly suggestive in risk subjects. Ivermectin is generally regarded as the most effective therapy [109, 119].

## Dracunculiasis

### The parasite, its cycle, and human infection

Guinea worm disease or dracunculiasis is a subcutaneous disease caused by Dracunculus medinensis. Eradication programs have successfully reduced the incidence and prevalence of this disease and it is now confined to a few sub-Saharan African regions. In 2015 only 22 cases of dracunculiasis were reported [120–122].

Dracunculiasis is transmitted to humans through drinking water contaminated with cyclops, a copepod (water flea) that acts as intermediate host and carries larvae of the worm. About a year after a person has become infected, adult female worms emerge from the skin (usually one to three emerge simultaneously). If the emerging worms make contact with water, they expel larvae into the water, which the copepods ingest, beginning the cycle anew [120, 123].

Developing worms do not usually cause symptoms, but as Guinea worms emerge, they cause burning pain and may provoke allergic responses including urticaria or even asthma. Abscess formation due to bacterial superinfection is common. It usually affects legs and feet. Worms migrating near a joint sometimes cause arthritis with joint effusion [109, 124].

### Imaging findings

**Fig. 16:**
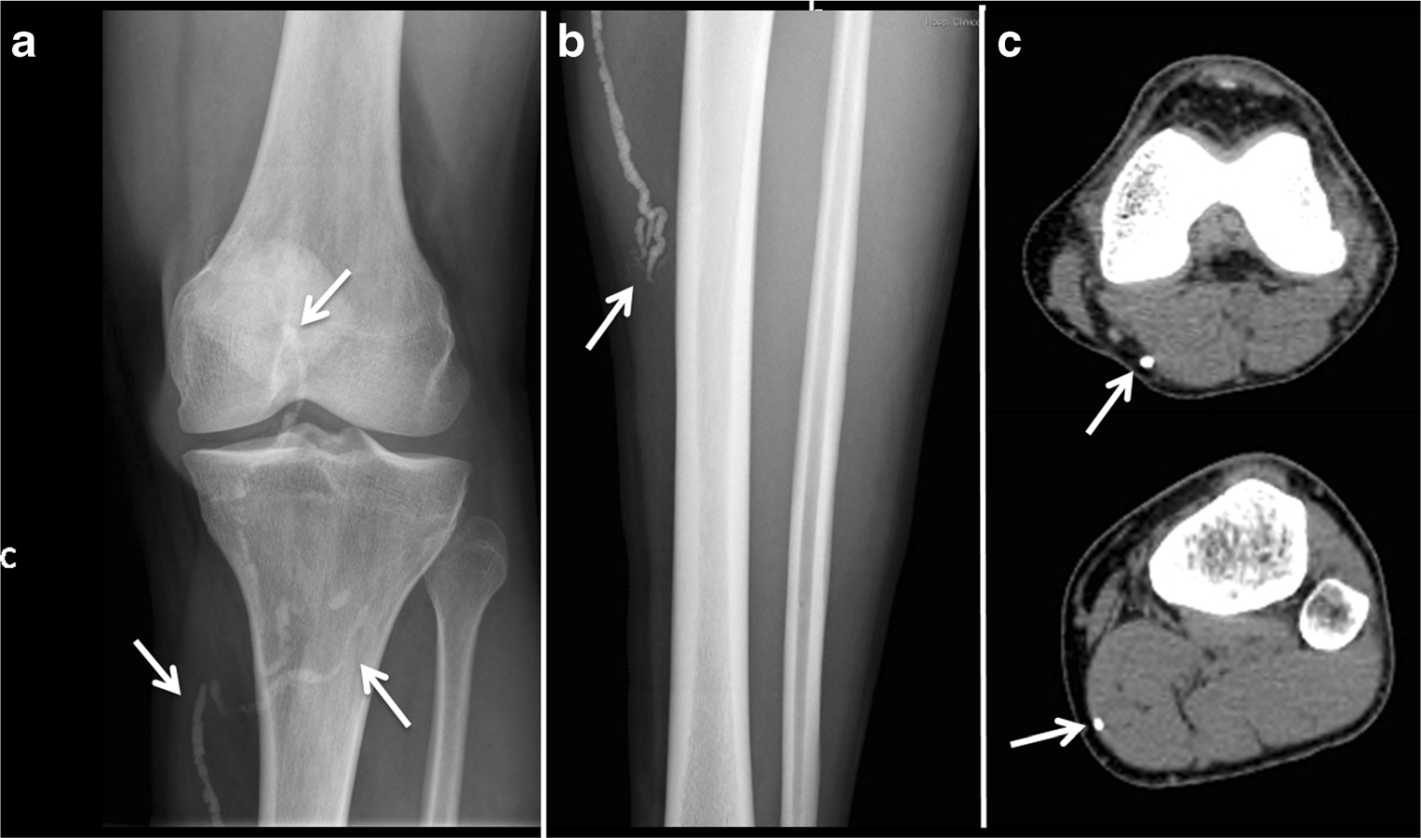
Dracunculiasis. **a** and **b** Plain radiographs of the left knee and leg of a patient coming from Mali shows “worm-like” calcifications in the soft tissues caused by dead calcified worms. **c.** CT image of the left leg of the same patient as in **a** and **b**, confirms the dead calcified worms in the subcutaneous tissue and muscles of the left lower limb

Dead calcified worms can be seen, many times incidentally, as soft tissue “worm-like” calcifications on radiographs or CT (Fig. 16).

### Diagnosis and treatment

Diagnosis is mainly clinical. The traditional method to discharge the larvae is to tie the end of the emerging worm to a stick and wind the worm out slowly. There is no effective anthelmintic agent. Provision of a safe drinking water supply is the key to fighting this disease [109, 120, 125].

## Anisakiasis

### The parasite, its cycle, and human infection

Anisakiasis is caused by the consumption of raw or undercooked fish containing larvae of the anisakis worm.

Anisakis species have a complex life cycle with several intermediate hosts (different species of crustaceans, fish, or squid), and a final host, which are big sea mammals (whales, dolphins, or seals). Humans are accidental hosts of the parasite. Anisakiasis has a higher incidence in areas where raw, marinated, or pickled fish is commonly consumed, i.e. Japan, Korea, Latin America, and Europe (in particular Scandinavia, the Netherlands, Spain, France, and Britain) [126, 127].

After ingested, anisakis larvae stuck on the wall of the human gastrointestinal tract unable to penetrate it. Depending on the site where the larvae attach, anisakiasis can be divided into gastric, intestinal, and ectopic, the latter uncommon [128, 129]. Stuck anisakis larvae cause direct tissue damage in the bowel wall and trigger an inflammatory and allergic reaction leading to the formation of eosinophilic granulomata. Ulceration and perforation of the wall can also occur [126, 130].

Gastric anisakiasis is more common than small or large bowel anisakiasis [128, 131], and acute abdominal pain, nausea, vomiting, and fever are its major symptoms [128, 132]. Intestinal anisakiasis can manifest with abdominal pain, diarrhoea, peritoneal irritation, ileus of the small bowel, or even bowel obstruction. Intestinal anisakiasis of the distal ileum can mimic acute appendicitis or Crohn’s disease [130, 133]. An acute systemic IgE-mediated allergic reaction may occur as well, including anaphylaxis [126].

### Imaging findings

US and CT are useful techniques to suggest the diagnosis of intestinal anisakiasis, which is often clinically under-recognised due to the long interval (commonly 1 week) from the intake of contaminated food to the onset of symptoms. This differs from gastric anisakiasis, which develops symptoms a few hours after the ingestion of the larvae [131].

**Fig. 17:**
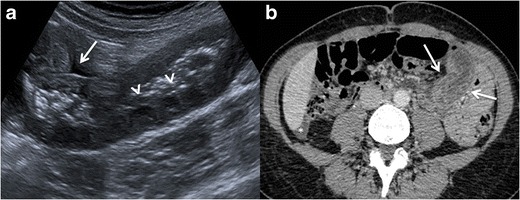
Anisakiasis. 44-year-old woman presenting with severe abdominal pain four days after having eaten raw fish. **a.** US scan axial image, concentric wall thickening in several jejunal and ileal loops and ascites (*arrow*) were seen. Note the irregularity of the lumen surface due to oedema of Kerckring’s folds (“the corn” sign) (*arrowheads*). **b.** Contrast-enhanced CT of the same patient as in **a** shows diffuse concentric wall thickening of a jejunal loop (*arrows*) and ascites (*asterisk*). The patient had positive test results for specific IgE anti-anisakidae antibodies

Typical US and CT findings of gastrointestinal anisakiasis are severe submucosal oedema of the involved segment of the gastrointestinal tract and ascites [126, 132, 134]. US typically shows marked oedema of Kerckring’s folds, which is known as “the corn” sign (Fig. 17) [128, 135].

### Diagnosis and treatment

In gastric anisakiasis, the anisakis larvae are frequently found on endoscopy attached to the stomach wall [128]. In contrast, in intestinal anisakiasis, the diagnosis is commonly made with a combination of clinical history, the positive results for anti-anisakidae antibody and the presence of the characteristic intestinal lesions on imaging techniques (Fig. 17) [126, 128].

The treatment of gastric anisakiasis is either endoscopic removal of the parasites or conservative management. Intestinal anisakiasis is generally treated with conservative management. However, there have been cases of strangulation or severe long segmental stenosis of the intestine caused by anisakis, which required surgical treatment [128, 134, 136–138].

## Conclusions

Parasitic diseases are seldom encountered in our daily practice in Europe, but we should keep them in mind and be familiar with their main imaging findings, especially in the adequate clinical context, as they are emerging conditions due to immigration from endemic areas and also some of them are still endemic in certain European regions. It is our mission as radiologists to recognise these diseases and raise the suspicion in order to promptly diagnose and manage them.
